# Wishes to die at the end of life and subjective experience of four different typical dying trajectories. A qualitative interview study

**DOI:** 10.1371/journal.pone.0210784

**Published:** 2019-01-17

**Authors:** Kathrin Ohnsorge, Christoph Rehmann-Sutter, Nina Streeck, Heike Gudat

**Affiliations:** 1 Hospiz im Park, Hospital for Palliative Care, Arlesheim, Switzerland; 2 Institute for History of Medicine and Science Studies, University of Lübeck, Lübeck, Germany; 3 Institute of Biomedical Ethics and History of Medicine, University of Zurich, Zurich, Switzerland; Foundation IRCCS Neurological Institute C. Besta, ITALY

## Abstract

**Research aims:**

The motivations that lead to wishes to die (WTD) in palliative care patients with cancer are relatively well studied. But little is known about WTD in other pathologies and the relation between subjective understandings of dying trajectories and a WTD. We investigated the WTD of palliative patients in four different dying trajectories: neurological diseases, organ failure, frailty due to age, and cancer.

**Study population:**

62 palliative cancer (n = 30) and non-cancer (n = 32) patients (10 neurological disease; 11 organ failure; 11 frailty), their families and health professionals in different palliative care settings (248 interviews).

**Study design and methods:**

Qualitative semi-structured interviews. Data analysis through Interpretive Phenomenological Analysis and Grounded Theory.

**Results:**

In addition to personal motivations, we found that people dealing with similar trajectories were often confronted with similar questions and concerns due to similar challenges. For four trajectories we show typical patterns, similarities and differences that should be considered when talking with patients about their WTD. These illness-related considerations do not explain the WTD completely, but give important information on the challenges for particular patient groups that might experience a WTD. In all patient groups, there were clear moments that triggered a WTD: for neurological patients it was experiencing breathlessness, high-dependency care, or when considering tube feeding or respiratory support; for persons with organ failure it was an acute burdensome crisis; for patients with cancer after the initial diagnosis, it was the first relapse or the move into advanced palliative care; for elderly frail persons it was the move into care facilities, or the loss of important relationships or capabilities. The feeling of being a burden to others was reported in all patient groups.

**Interpretation:**

WTD can be triggered within disease trajectories by specific conditions and transitional points that affect agency and self-understanding. A better understanding of the concerns and challenges of a particular dying trajectory as well as its characteristic trigger points can facilitate early and comprehensive communication about patients’ WTD, and the underlying motivations and protective factors.

## 1. Introduction

Research shows that wishes to die (WTD) appear relatively frequently in patients undergoing end-of-life care (7–40%) [1]. These wishes often have multidimensional reasons, hold subjective meanings, and consist of diverse and dynamic intentions, which in addition are weighed against each other over time [2][3][4]. A WTD does not always contain a wish to hasten death [5][6][1]. It is not uncommon for patients’ wishes with regard to living or dying to change from WTD (and even wishes to hasten death) into wishes to live and also the other way around, even in the final weeks of life [7][8][9][10]. All this might suggest that for many patients, thoughts of dying and even wishes to die, transient or not, might be part of a process of coming to terms with their situation at the end of life, of facing loss, suffering and finitude. For several reasons, patients often face difficulties in talking frankly about these thoughts or wishes to health professionals. However, not addressing these ideas can lead to isolation and eventually to physical, emotional or spiritual needs not being met [11]. Hence, we believe that regular sensitive invitations to communicate and explore patients’ needs and wishes–if patients so desire–should be standard in end-of-life care.

In recent years, a large body of research has been published that gives insight into the etiology and subjective experiences of WTD and communication around these wishes [11][12]. Much of the research has either been done with cancer patients, or without distinguishing pathologies. Relatively little is known about WTD in non-cancer patients (with the exception of some patient groups, such as people with amyotrophic lateral sclerosis (ALS) or the frail elderly). We can only speculate about whether or in which way the different diseases’ associated trajectories of dying [13] influence the subjective experience of WTD. We can hypothesize that the particular challenges in the subjective experience of different dying trajectories set the conditions against which WTD are articulated. Particular illness-related symptoms, or typical psychosocial concerns and experiences related to particular dying trajectories, might contribute to or trigger a WTD. But while there is some quantitative knowledge of the experience of different dying trajectories and the various types of distress and dependencies [14][15], we know of no studies looking at how terminally ill or elderly patients themselves experience particular dying trajectories in relation to their WTD. More in-depth knowledge could facilitate communication about end-of-life wishes and be helpful in assessing particular patients’ needs and fears more accurately and responsively. Hence, within a study of WTD in terminally ill and frail patients, we investigated subjective accounts of WTD statements in relation to experiences of different disease and dying trajectories. We did this in four patient groups that typify dying trajectories in palliative care [16]: persons with cancer, neurological diseases (ALS, MS), organ failure (heart, lung), and elderly, frail persons with multiple pathologies. Here we present results on the interrelation between subjective understandings of dying trajectories and the WTD.

## 2. Overview of the literature

### Incidence

The incidence of WTD is best characterized among cancer patients. It is known that 7.7% to 10% of cancer patients express a WTD [17][18]. Research results vary greatly, however, depending on how the ‘wish to die’ is defined. The prevalence of WTD in terminally ill cancer patients ranges from 44.5% of patients with a moderate desire to die [1], to 8–17% with a more persistent WTD [17][18][19], and 10.6% who express a serious desire for assisted dying [20]. In a Canadian cross-sectional survey of 377 cancer patients, 69.5% had no desire to die, 18.3% expressed occasional transient thoughts, and 12.2% said they had a genuine desire to die [21].

In persons with neurological diseases such as amyotrophic lateral sclerosis (ALS) or multiple sclerosis (MS), WTD statements seem to be more frequent. A study in Switzerland and Germany [22] showed that 44% of ALS patients interviewed had already thought once about suicide, every second patient could imagine asking for assisted dying, and 14% currently had a WTD. This corresponds to other international findings in the quantitative literature [23][24][25][26][27][28]. Only one study found that wishes to hasten death was low in this group and even decreased within one year despite physical decline [29]. In comparison, it is reported that 10–20% of elderly persons express a WTD [30][31][32][33][34][35]. Patients with organ failure seem to be comparatively under-researched. A Dutch questionnaire-based study [16], in which physicians reported on patients with different pathologies who made a request for euthanasia or PAS, refers to 0.5% of all heart failure patients dying from PAS compared to 5% of cancer patients and 20% of ALS patients in 2005.

### Motivations for wishes to die

In cancer patients, pain is a frequent, important symptom, and patients mention it as a reason for their WTD [14]. Pain and fatigue are also reported as the most important causes of unbearable suffering, leading to a request for euthanasia in cancer patients [16]. However, under palliative care and with adequate pain control, pain is less frequently stated as a reason, and the motivations given for a WTD include existential-spiritual and non-physical ones [12][17] such as depression, demoralization, hopelessness, a sense of abandonment, fear of the future, fear of losing control or the sense of self, the perception of being a burden to others, poor family cohesion or social support, high levels of anxiety, and existential suffering [36][37][38][39].

ALS patients gave fear of suffocation and dependence as the main causes of unbearable suffering leading to a WTD [15]. While heart failure patients most frequently gave somatic issues (especially pain and fatigue) as causes of unbearable suffering, ALS patients reported less somatic and more psychosocial motivation than other patients. Hopelessness and end-of-life despair, much more than depression, are frequently discussed as important triggering factors underlying a WTD in ALS [40][41][42] (for hopelessness in ALS in general see [43][44]). The findings of the Dutch research [15] support those of other studies, which identified the fear of being a burden to others as an important triggering factor for a WTD in ALS patients [29] [40]. The fear of dependency on others as well as becoming immobilized were also mentioned frequently by all patient groups (ALS, heart failure, frail elderly) [15].

In elderly patients, the WTD is associated with being female, not being in a partnership, greater frequency of depressive symptoms, loss of autonomy and controll, financial problems, restricted social network, urinary incontinence, a negative perception of one’s own physical health, polymorbidity, higher levels of stress and sleep problems, and living in a nursing home [30][45][46][47][48][49][50]. In the elderly, loneliness and social disconnectedness in particular play an important role in WTD, while the presence of social support can effectively counteract a WTD [9][49][51][52]. Untreated or undertreated depression might cause a WTD, and while most authors agree that underdiagnosed depression in the elderly is a problem, clinical depression is not always present in elderly people with a WTD.

Persons with heart failure state the experience of dyspnea, dependency, and the knowledge that suffering will get worse as the main causes of unbearable suffering leading to a WTD [15]. The disease trajectory of organ failure is typically characterized by an up-and-down of crises and subsequent stabilization at a slightly lower level of physical wellbeing, which makes prognosis in palliative care difficult (which crisis will be the final one?). Research depicts these patients as trying to live “daily life as usual” and because of that trying “to deny the threat to life for as long as possible” [53]. Because of prognostic uncertainty and so as not to destroy patients’ hope and motivation to overcome the next crisis, it is well known that doctors tend to avoid talking to patients about their prognosis [54][55][56]. “Prognostic paralysis” [57] is believed to impede open communication, and advanced care planning and is known to cause delayed and significantly reduced enrolment into palliative care (7% of all patients with heart failure versus 48% of all cancer patients in primary care enrolled in palliative care in the UK in 2009 [54]).

## 3. Method

Our method relies on a phenomenological-hermeneutic framework and is based on Interpretative Phenomenological Analysis (IPA) [58]. The idiographic approach of IPA enables the in-depth investigation of how persons make sense of and attribute meaning to their individual experiences. Such an approach has the advantage of studying in depth the individual person’s thoughts, wishes and actual experiences in the final phase of life, and allows insights into the complexity of their WTD. We conducted and analyzed qualitative interviews with patients and their caregivers, in order to investigate wishes to die or to live from the subjective perspective of terminally ill or elderly persons in palliative care. The results that we present here derive from one of the main categories of analysis (‘WTD and subjective experience of dying trajectories’). In order to compare the data between the different patient groups we then applied an analytic tool (derived from the ‘anatomy of a WTD’ described below) that we developed in the first part of the study, which involved 30 cancer patients, their physicians, nurses and relatives, and was based on a grounded theory approach [59, 60].

Sampling: For each patient, we also interviewed a close family member (with the patient’s consent), and always a physician and a nurse (total interviews n = 248). For the detail of the sample see Tables 1 and 2.

**Table 1:** Patients’ characteristics I.

	Cancer	Non-cancer
Number of cases	30	32
Age	34–87 Median 82	58–97 Median 82
Sex M/F	12/18	18/14
Deceased	28	11
Interval between last interview and death (days)	5–237 Median 23	3–480 Median 53
Depression mild to moderate	3	4*
Interviews with family caregivers	18	20
**Total interviews**	**116**	**132**

*In 3 out of 4 patients depressive mood overlapped cognitive impairment

**Table 2:** Patients’ characteristics II.

Cancer		Non-cancer	
Pancreas	5	Organ failure	11
Breast cancer	5	Neurological disease	10
Digestive tract	5	Frailty	11
Prostate	3		
Lung	2		
Ovarian	2		
Other (chordoma, glioblastoma, lymphoma, liver or bladder carcinoma, primary tumor unknown)	8		
**Number of interviewed patients**	**30**		**32**

The patients were situated in a variety of palliative care facilities: a hospice, a palliative ward, specialized units in acute care hospitals, a clinic for neurological diseases, several nursing homes, or outpatient palliative care. Where possible, we conducted more than one interview with the patient. The patients interviewed satisfied the following inclusion criteria: persons (a) with incurable and advanced tumors, neurological disease, organ failure or frailty/old age, (b) in a palliative situation (defined by the Gold Standard Framework (http://www.goldstandardsframework.org.uk/), (c) who were already aware of the incurability of their condition or palliative state, (d) whose primary physician had consented to their enrolment in the study (patient protection), (e) who had sufficient language skills to participate in the interview, and (f) who were cognitively in a condition to participate in the study.

For the informed consent procedure, patients were approached by their physician and informed orally and through an information brochure about the study (see S1 and S2 Supporting informations. Patient information sheet in German and English). Patients expressing interest were contacted by the research team. The interviewer answered participants’ questions and obtained written informed consent before the interview started (see S3 and S4 Supporting informations. Patient informed consent form in German and English). For some interviewees with advanced neurological diseases who were unable to sign the informed consent, we audiotaped oral informed consent at the beginning of the interview. Written patients’ informed consent was also requested for the collection of information from medical records. Strict confidentiality between interviews was guaranteed. For interviews with relatives, we asked the patients for their agreement and, if they agreed, to indicate which of their relatives they wished to be interviewed. Relatives and healthcare professionals were contacted by the research team, and gave written informed consent before the start of the interview.

Each patient was tested for depression (anamnesis and screening by Robinson’s mini-screen for depression [61] and the Beck Depression Inventory in suspected depression [62]), although the existence of treated depression was not an exclusion criterion. We deliberately did not restrict inclusion to patients who had previously expressed a WTD, because we also wanted to investigate the genesis of WTD or situations in which a WTD was present but not known to others. We deliberately did not investigate wishes to hasten death (WTHD) alone, but wishes to die (WTD) in general, as we believe that there are various forms of WTD and that expressions of WTHD should be situated and interpreted within the larger context of patients’ current wishes and concerns.

Participant enrolment happened in parallel with the ongoing process of data interpretation. At the outset, we included patients broadly, with or without known WTD according the criteria mentioned above. In the course of the study, we chose interviewees increasingly more selectively, involving more persons who had already expressed a WTD. The first part of the study focused on WTD in cancer, while in a second phase we looked at dying trajectories in non-cancer patients. Of all patients included in the study (n = 62), 30 had cancer. The other 32 fell into three groups: neurological diseases (mainly ALS and MS; n = 10), organ failure (especially lung and cardiovascular; n = 11), and frail elderly patients (n = 11). For patients’ characteristics see Tables 1 and 2. Both study phases (cancer and non-cancer patients), which are evaluated jointly here, were approved by the Ethics Committee of Basel (EKBB).

The semi-structured, face-to-face interviews lasted between 30 and 120 minutes and focused on the experiences, ideas and wishes of the patients with regard to their dying. The interviewers were not involved in the care of the interviewees; KO, NS, HGK and two other interviewers (Lucia Stäubli, an art therapist in palliative care and Heidi Gass, a palliative care nurse) were either trained and/or experienced from the pilot and first interview study, or took university courses in qualitative research. KO, NS, CRS and HGK were part of the main research team and took part in the interpretation of the transcripts. An interview guide and a detailed description of the methodology can be found in the appendix to [2]. A preliminary version of the interview guide and the analytical methodology were tested in a pilot study and continued to be refined throughout the entire interview period. A major revision took place after the completion of the first part of the study with 30 oncology patients. Interviews with nurses, doctors and relatives took place shortly after each patient’s interview, and were based on an adapted interview guide focusing on the patients’ experiences, i.e. they explored these individuals’ perception of patients’ experiences, views and wishes with regard to dying, and the communication about them. All interviews were audiotaped and fully transcribed by two transcribers who were not involved in the interview process but had been trained through the pilot and first part of the study on cancer patients. The medical records helped to characterize the medical circumstances in which the patient developed his or her wishes.

For the analysis, we adopted a case-by-case approach. The transcripts of each patient with the accompanying transcripts of the family and professional caregiver interviews were first read and open-coded by each of the authors independently. Analysis was supported by MaxQDA11. The emerging themes found by individual analyses in each case (consisting of the patients’, the family’s and the professionals’ interviews) were then discussed by all authors in interview interpretation sessions. In-depth discussion of individual interpretations in the research group helped to clarify areas of interest and possible preconceptions [58] and usually lasted until we reached a shared interpretation of each case. The analysis led to the elaboration of emerging themes and the selection of the most important themes, set by the interview guide and the underlying research questions, first for each case and in a second step by comparison between cases. We continuously compared cases in terms of the set themes (those defined by our research questions) and emerging themes. Several emerging themes were selected and investigated in depth over the study period and the entire data set.

**Fig 1:**
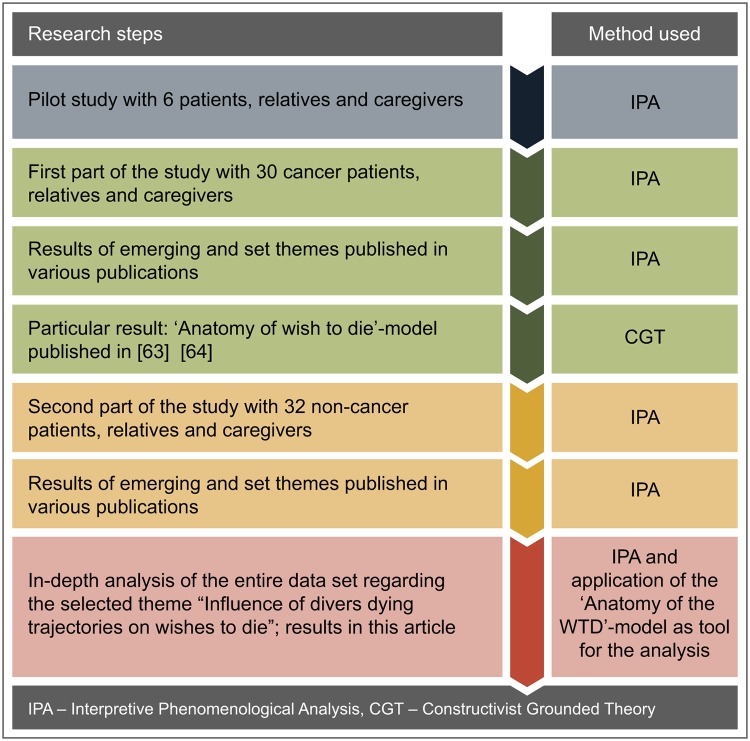
Research steps and methods used over the entire research period.

This article is based on our findings about the ‘influence of diverse dying trajectories on the WTD’. For this analysis, we used a theoretical model (the ‘anatomy of a WTD’ model) that we developed during the first part of the study on cancer patients (see [63][64]). The development of this model from the data was based on a constructivist approach to grounded theory [60] (see Fig 1).

The ‘anatomy of the WTD’ describes three different dimensions that can be investigated in order to understand the meaning of a WTD for a patient. These dimensions are: a) the intention of the WTD: what this wish aims for; b) the motivations behind a WTD: why the wish is there, i.e. subjective reasons, meanings or functions of the WTD for the patient; and c) the social interactions that might influence a WTD. For this publication, we applied these three dimensions as an analytic tool to investigate the similarities and differences in WTD between the different trajectories. We then wrote outlines of characteristic patterns for each trajectory group. In order to identify and highlight the differences, we extracted quotations about the ‘influence of diverse dying trajectories on the WTD’ and ordered the emerging themes under the superordinate themes given by the ‘anatomy of a WTD model’. This enabled us to compare the data of the second part of the study on non-cancer patients with those from cancer patients. In doing this, we especially looked at the differences that correlate with the circumstances of the different disease groups. We were aware of potential shortcomings when comparing data in different disease-related contexts [65].

In the general analysis, we took the interviews of relatives, physicians and nurses first as independent perspectives, but then investigated the interactions of the family and professional caregivers with the patient in terms of the WTD and the co-construction of knowledge and wishes: what do they know from each other? How do they communicate? How does communication happen? Which moral values, concerns or expectations influence the interaction and the WTD, and whose are they? How do people react and counter-react, and how does this shape the WTD? etc. In this paper, however, we focus primarily on the patients’ perspective and on the question of whether and how their subjective experience of illness shaped their WTD. The co-constructive aspect of the WTD is represented here mainly in the sub-theme of ‘not wanting to be a burden to others’. In other papers [2] [7], we have highlighted how WTD are shaped by social interactions between patients, their families and their professional caregivers.

In addition to individual memos made during coding, we prepared theoretical case memos based on the discussions within the research group for each case analysis and recorded the team’s interpretation of important key themes. We also prepared memos of the final analysis of the trajectories and according to the above mentioned ‘anatomy model.’

The present paper originated from one of the initial research questions: Do the different dying trajectories differentially influence the WTD, and if, so how? This paper presents our comparative findings from all four groups investigated in terms of the three dimensions of a wish to die represented in the WTD model, focusing particularly on how the patients’ subjective experience of their illness and dying influences their WTD.

## 4. Results

WTD express very personal concerns and processes of confrontation with one’s own death. In these processes, individual experiences, personal moral understandings, relationships and cultural issues play an important role [3][63]. However, in the subjective accounts of persons going through the same illness or frailty due to very old age, we found that interviewees explaining the presence of their WTD expressed recurring considerations and experiences connected to the particular illness or health status alongside other more individual concerns. These considerations do not explain the WTD completely, but give important information both about the challenges for particular patient groups that might lead to a WTD, and what to investigate in more detail once a WTD is experienced. This information sometimes differs from third-party observations or more objective descriptions of the dying trajectories [13]. What we present here as ‘trajectories’ refers to the similarities we found in the subjective experiences of people with the same illness in terms of their explanation of their WTD. In the introduction to each subgroup we describe general features of the experience of a particular illness as reported by our interviewees (patients). This description is based on the entire data set of patients with the same illness. The results for the WTD associated with this particular illness, however, are based only on the patients who expressed a WTD.

### 4.1. Wish to die and the experience of dying in patients with neurological diseases

Our study included 7 patients with ALS, 2 patients with multiple sclerosis (MS), and 1 patient with inclusion body myositis. All patients were aware that their progressive disease would, in the foreseeable future, present them with challenges and circumstances of dying that would require them and their families to make major decisions. Most of these people were able to rely on a well-established web of healthcare support and advanced care planning with their professional caregivers. All the ALS and MS patients had been told by their healthcare professionals that they would need to decide at some point in the future about starting artificial nutrition and hydration or respiration support. Depending on the support and quality of information given by their healthcare professionals, they knew about the medical care support they could count on. Many of our interviewees with neurological disease were concerned with the question of how dying could happen in a dignified way under these particular circumstances. Their ideas about dying were often shaped by these concerns. Patients felt supported by contact with other patients or with self-help groups that were offered by the competence center.

#### Intentions

All six persons with neurological disorders who had a WTD formulated a *hypothetical* WTD (i.e. not for the present moment but for the future, in a situation when life became unbearable for them). One person, at the moment of the interview, had a strong desire to die but without wanting to hasten death. He said: “Whether I’d have the courage [*pause*] to kill myself, I don’t know. But I would genuinely like to fall straight down dead.” (P5-II). (For the larger conversational context for all quotes in the German interviews, see S5 Supporting information. Original German quotes in relevant interview context). A single male patient (P32-II) made concrete plans to hasten death through a right-to-die organization. Others said they had long been members of a Swiss right-to-die organization and considered contacting them in future; however, these choices seemed farther away for them. Four had temporarily experienced an active wish to hasten death during a moment of crisis (P5-II, P8-II, P9-II, P34-II). For example, during a respiratory crisis one woman with advanced ALS reported asking her husband for a barbiturate to end her suffering: “Then [during the acute respiratory crisis] I said to my husband, now I want a tablet of some sort and pff! You can’t get them [tablets/barbiturates].” (P8-II). However, most of these patients, except P5-II, 67 years old, who had had MS since adolescence, said that they had moments or aspects of life they also enjoyed, and expressed a wish to live alongside their WTD.

#### Motivations

Patients with neurological diseases explained their WTD in terms of their worries about future situations or conditions they believed would be too burdensome. They were very aware that these situations would probably occur as part of terminal illness. Mostly, they feared being entirely paralyzed, dependent on external respiration, having pain, or not being able to talk, walk or turn over. For example, a woman aged 66 (P8-II), with first manifestations of ALS 6 years ago including swallowing disorder, esophageal spasm and dyspnea, stated in the interview:

“Yes, I think, after a certain point there won’t be any more alternatives (…) if even every breath is a burden, so when you can’t move in bed on your own, I think, there are situations where that becomes simply unbearable. (…) [if you] can’t eat any more, can’t speak any more (…) well, then you lie there and get sore from lying, they turn you, hardly have you been turned when–ah–everything hurts.” […] “Then I would say the point has come when, I just have to [say that it] isn’t life any longer. […] And if you then help it along a little, I think that’s legitimate (…)”

(P8-II)

It was not clear what she meant with “help it along a little”. Assisted suicide was not an option for her.

Total dependence and the “image of a total loss of sovereignty” (son of P32-II) was expressed as their greatest fear by nearly all of our interviewees with neurological diseases. Some of them also feared being a burden to others. A patient (P32-II), 78 years old, widowed and living alone, with initial ALS manifestation 1.5 years ago and rapid muscle wasting, considered hastening death through a right-to-die organization when he had to leave his home. If he were totally dependent, life would lose meaning for him: “If I’m no longer free and can’t do anything on my own, I’m totally dependent, then I would probably feel that my life didn’t have much meaning anymore.” (P32-II). But he also explained his WTHD by saying it would be important to him for his family and friends to remember him in a state where they could communicate with him: “And I don’t really want to waste away miserably. But to leave this world in a state where I can remain somewhere in the memories of my children and my friends and so on as someone who, well, you could talk to and who was reasonably normal.” (P32-II) He died five months later in the emergency ward of an acute hospital from respiratory decompensation.

One male patient, who suffered from advanced MS, was a wheelchair user and lived in a long-term care institution, with his daughter as the only person caring for him outside the institution. He explained his acute WTD (without wanting to hasten death) by his experience of the radical loss of independence and decreased quality of life. His statement resounds with a sense of loss of dignity, and shame about his situation:

“How it is now, that’s just, for all of it, for wiping your bottom, to pee you need a bag… You just always need help for everything. Recently we went to the council, to sort that out. So that my daughter gets power of attorney, because of the signatures, yes? I can’t sign things anymore! I can’t even do that!”

(P5-II)

Some of those who had a hypothetical WTD for the future explained that this wish served a function for them. They said that it gave them the feeling of still being in control of their lives when confronted with increasing loss of autonomy. Some of them said that they were members of a right-to-die organization, so as to be sure of “keeping the last door open” to be able to decide for yourself (P34-II). Thinking about the option of hastening death in future expressed a need for the security of having an option of last resort should the situation became unbearable. Advanced care planning was therefore also extremely important. Some said that an open discussion with their physicians about the prognosis and about healthcare options, including palliative sedation and the reassurance that they would not have to suffocate, was extremely important to them in counteracting a WTD.

#### Social interactions

Several social aspects influenced what people with a neurological disease wished with regard to dying. Observing the suffering of other people with the same illness often brought people with ALS or MS to wonder what kind of development, symptoms or state of health they themselves would find unbearable. Sometimes these observations gave rise to the expression of a hypothetical WTD. One man with MS, remembering a friend with MS who spent months in a state of dementia in which she did not recognize anyone, said: “No. I mean, if I realized that something isn’t good any more like this [i.e. the dementia in his friend with MS], then I wouldn’t want to live any longer.” (P32-II)

Some neurological patients with a WTD told us they were worried about being or becoming a burden to family caregivers due to increasing dependence and the high need for care. Most patients were aware of the impact of care on their families. One woman suffering from ALS, and mentioned above (P8-II), explained to her husband that she would want not to be resuscitated, so as to spare him and herself years of suffering in which he would have to care for her: “because [*breathes*] there would be years of suffering ahead, for me and for you, I said, because he has to look after me, he isn’t free either then. It took me a long time to make him understand it, but I think it got through.”

However, more often, interviewees told us that strong family bonds and loving relationships were perceived as supportive, and counteracted a WTD (P5-II; P9-II; P32-II II 30ff). Several patients told us that even if they had a WTD they would not take their own life, because they did not want to inflict more suffering on their loved ones, or simply because they wanted to enjoy time with them as long as they could. In the course of their illness, some experienced their families as counterbalancing their weakening physical function and saw this as supporting a strong sense of belonging that deepened during the experience of illness. One man who lost his voice due to ALS and who refused electronic speech support relied on his wife to ‘translate’ what he formulated (‘my wife is my voice’) (P7-II).

However, there were also stories of relationship breakdown, pre-existing couple conflicts, and families who experienced major tensions because of illness. One patient expressed a WTD at the point when his wife told him he had to move to a nursing home within six months. The blunt conversation, and the idea of being sent to a nursing home, made him think “that I would be shut away, like putting the rubbish out.” (P15-II) At the time of his wife’s decision she had been caring for him for at least 8 years, and in the previous 2 years he suffered from complete spastic tetraparesis and aphonia, communicating via tablet. For her it had been full-time, round the clock work. The strain of caregiving over such a long time and continuous conflicts in communication between the couple (possibly also provoked by changes in his personality due to the illness) added to pre-existing couple conflict.

### 4.2. Wish to die and the experience of dying in patients with organ failure

People with organ failure experience their dying in the context of a disease with a progression associated with ups and downs, crises followed by stabilization at a slightly lower level of functioning. These patients told us that the continuous and repeated demand to cope with recurrent health crises required enormous energy and motivation from themselves and those around them. Research often reports that these patients stay strongly focused on survival and overcoming the next crisis, and they (as well as their healthcare professionals) have difficulty acknowledging the proximity of dying [53, 57]. However, our study patients were undergoing intensive medical treatment. According to clinical assessments, as the Gold Standard Framework, they all were in their final period of life. They complained that the phases of stability between crises were shorter, their physical function was decreasing, and most of them were clearly aware that death was coming nearer.

#### Intentions

Some patients with organ failure and WTD (P6-II, P11-II, P20-II, P26-II) made an active request for help to hasten death during an acute crisis, others because of a significant deterioration in their health (P29-II). Like the other groups, their WTD were dynamic and were continuously weighed against the wishes to live that were also strongly present for most of them. After the experience of an acute life-threatening health crisis, however, alongside their wishes to live many of these patients also developed a hypothetical WTD for the future, wanting to avoid having to go through another crisis.

Some patients had more concrete plans to hasten death that were important to them for at least a period of time: a woman with pancreatic insufficiency (P11-II) first contacted a right-to-die organization and asked them to reserve a prescription for the barbiturate (the common procedure of these organizations). Under slow recuperation but still in weak health, her acute WTHD turned into a hypothetical WTD in the event of another crisis. Another patient with cardiac insufficiency (P26-II) mentioned that he had made a suicide attempt by excessively drinking water to provoke a stroke, after his urologist had explained this risk of complication. Subsequently he agreed with his physician to concentrate on palliative support only and to refuse any further curative treatment. But there were also some patients who wanted to fight to overcome the next crisis until the very end (P2-II).

#### Motivations

Patients reported that coping with a life-threatening crisis required enormous amounts of physical and psychological energy. Some explained that they had already accepted that they were ‘dying’, and found it difficult to readjust and continue living. The burdensome experience of previous crises (e.g. acute respiratory crisis) and the fear of having to go through another made some patients consider the possibility of hastening dying, and some then decided to let death happen at the next crisis.

What some patients with organ failure found particularly stressful was that they felt as if they were ‘hanging between life and death’. They wanted to be able either to live or to die, but not to live with recurrent crises at ever shorter intervals, a life that some described as an ‘intermediate state’: “I said, I can’t stand this: either die or live, but not this in-between thing.” (P11-II).

Asked about the reasons for her acute WTHD, the same woman said that she had a hard time imagining engaging in life again:

P: If I have a relapse, a bad one, then I’ll do it [*assisted suicide*]. Then I’ll set it in motion. That’s my son, he’ll set it in motion.

I: So what happened, to make you decide you want to die now, you want to go to EXIT[*right-to-die organization*]? What triggered that?

P: Because I thought, I can’t bear it, starting life over another time. Going through it all again. (P11-II).

Others explained their WTD by saying that they perceived treatment as too stressful or that it would reduce the quality of their life too much. An 80-year-old man experienced the hours of dialysis not only as extremely “boring”, but said they prevented him from participating in significant social activities in his nursing home, compromising his quality of life until the point that he sometimes wished to die. Another man (P26-II) found his multimorbidity becoming so complicated that it led to a situation in which the treatment options for particular symptoms mutually excluded each other. He described this as very tiring and as forming the background to his WTHD:

“You know you come to a point, as I said, where you run out of puff… And afterwards I’ll get heart problems, later on. And then they told me, I shouldn’t drink so much anymore. And previously, with my prostate, it was: ‘Drink lots, it’ll wash the stuff out!’ Because, I always had blood in my water, from time to time, didn’t I. And that was the clincher. So I said: ‘Then I’ve got a problem! If I don’t drink anything, down below. If I drink too much, up above. How am I supposed to do that?’ Then my doctor said: ‘Yes, that will be your problem. In future.’ Won’t it? And then you start to ponder and think: Why? You could also do it like this. And so it’s simple: we would like there to be a bit of reality, if possible. Everyone has to die. Up to now I haven’t found anyone who doesn’t *(blows his nose)*. That’s why I say–so–you can be for it [WTHD], you can be against it [hastening death].”

(P26-II)

#### Social interactions

Patients who reported having expressed a strong WTD to their physicians during an acute crisis said the physicians rejected it at that moment and did not accede to their requests to withdraw therapy, for example. These physicians held that such a decision required thorough communication and longer reflection than was possible in a state of crisis. One person who came to the emergency department with an infectious exacerbation of advanced COPD said that the physician ‘at that moment broke his will’ (P20-II), because he refused to follow his patient’s request to stop treatment. Retrospectively, however, the patient agreed with the physician’s decision. At some distance of time from the event, the same patient told us in the interview that he now had conflicting wishes regarding dying: on the one hand, he acknowledged that life was increasingly difficult and that he was close to death. But he said he had promised his wife to stay with her as long as possible. He also wanted to care for her as best he could. And he described himself as a candle, saying he was waiting “until the great wind blew him out” (P20-II).

Even though these patients lived with a great burden of illness and treatment, which often also caused significant life changes and demands on their family caregivers, the concern about being a burden to others was mentioned less often by our interviewees in this group than in other patient groups. This might have come through the small number of interviewed patients, but might be interesting to investigate in future studies with bigger samples.

### 4.3. Wish to die and the experience of dying in frail elderly patients

In the interviews, frail elderly people often described their life world as increasingly complex and wearisome: organizing the daily routine was becoming more and more “complicated”. Even small activities were more demanding. As mobility and concentration decreased, patients tended to organize everything well ahead of time: “Well, I always have to find out more about how I can manage all this. […] I used to be able to cope with it all myself. Just slowly.” P31-II. They were aware of being in need of increasing support and more dependent on others, which often resulted in their institutionalization in a care facility. Having been torn out of their familiar life world with its accustomed rhythms, they spoke about the experiences of alienation and loss, especially during their first time in a care facility. But even more than their outer world, they said they experienced their inner being as “complicated” (P30-I). What made their inner life complicated was often to do with changed thought patterns, as they frequently experienced recurring thoughts, past memories and forgetfulness. They often noticed a shift in or loss of their social roles, feeling less important and recognized by society. They saw their activities as less meaningful and happening more slowly than those of younger people. Taken together, all of this made them tired: “And I was just mentally overwhelmed. I’m not as strong as I once was.” (P31-II) Loneliness, alienation from the rest of society and the loss of meaningful activity played the most important roles here.

#### Intentions

Many frail elderly people experienced the loss of family members and friends, but also saw their own death coming closer. Approaching death was thus not an abstract event, but something they related to and pondered about, over and over again. As in the other patient groups, frail persons (28-II, 30-II, 31-II, 33-II) also expressed a variety of intentions in WTD, ranging from sporadically expressed WTD without any wish to hasten death (P28-II), to hypothetical wishes to die at some point in the future (P31-II), to concrete requests to hasten death (P30-II, P33, II). And like other patient groups, the interviewees often experienced a WTD alongside a wish to live, or the acceptance of dying. The increased frequency with which some of these interviewees repeated their WTD during the interview (especially for hypothetical WTD or WTD lacking the idea to hasten death) seems to be specific to this patient group. In some of these interviews the interviewees had circular or repetitive thoughts and the WTD was expressed repetitively.

#### Motivations

We hypothesized that the repetitiveness also served to acquaint the patients with and negotiate ideas and wishes about dying, to find their own attitude towards it, and to adapt to this final phase of life.

In addition, these expressions were also connected to other features of their experience of being old and frail, such as fatigue, loss of cognitive and other functions, and a level of decreasing interest in life. One woman frequently repeated that she would like to die soon, and during an acute pain crisis she even developed a WTHD. She said she wished to die because she felt overburdened by everyday activities and decision making and felt her inner life world was complicated: “I thought: well, now, now I can’t go on any more. I’m so finished. It’s all so complicated inside me.” (P30-II). She said that she had difficulties integrating the radical changes to personal life that came with old age, especially the continual losses of what was once important to her and to her self-perception. She compared getting old to ‘going backwards’ in personal development, which seems to imply a loss of capacities as well: “When you have […] to relinquish things piece by piece in old age, it’s difficult up here. In your head. That’s what [*bothers me*] most, it’s really a matter of life. […] If that’s life… you’re born. You have all these years. And when you go back, all that goes backwards again. And you have to experience so much where you think, no, that can’t be possible. […] It’s really not easy!” (P30-II) She also felt that nothing in her life really interested her any more: “I just hope that it doesn’t last much longer. It no longer really has meaning for me, you know. I’m not clinging to anything. […] I have no more desires. It’s here, but do what you want for all I care. It doesn’t mean anything to me anymore.” (P30-II)

Other elderly and frail persons did not feel at home, or disliked being in a nursing home, and said that this was the basis of their WTD: “Oh, I’d like to die now. I’d like to die now, and I hate the nursing home and the doctor said I should change [*homes*], but I can’t change any more. It’s too late. And it’s like this in every nursing home. Nowhere is it, nowhere is it, it’s more or less better, but um… um… it’s like this […] it’s not at all easy to exist in a nursing home.” (P31-II)

One of the most recurrent themes in a WTD of frail elderly people (and other patient groups) was shame about being dependent on others, and the fear of being a burden in a physical, psychological or financial way, as this woman who worried about being a burden to her children said: “I’ve constantly thought, now, ever since I wasn’t so well any more: Oh if only I could just die. Even if only because of the costs.” (P33-II)

#### Social interaction

WTD were significantly associated with changes in the perception or self-assessment of patients’ own social role. Some motivations mentioned above are linked to social interactions including assumed or real reactions of the surrounding world to the elderly that influence the way they wished to live or to die. Some of our interviewees felt their life was no longer socially significant and therefore worthless, feeling that they were not needed by anyone, or that they could no longer contribute meaningfully to the lives of others. Some underlined that since they had been socially active and cared for others all their life, the dependent life they lived now was particularly difficult for them–as this woman said: “But when you’ve just been there for others your whole life, and then you have to relinquish things piece by piece in old age, it’s difficult. […] well no, what am I here for actually, and the children are grown up, they have their own life, you don’t have anything to say any more. So I could just go, I think I could just go. […] I’m … I’m not needed any longer.” (P30-II)

Some frail elderly people associated their WTD with a fear of being a burden to others. One woman lived alone and had to call one of her children in an emergency during the night. She repeatedly stated that she wanted to die because she felt that her needs and those of her children clashed: “I called my son at one or two in the morning, whatever, for him to come and help me go to the toilet. That’s always very awkward, I did that for about two weeks. For two weeks I had to call on them at night without wanting to. She’s got a household and she [daughter in law] goes to work, and it’s very awkward for me.” (P33-II) In many comments the burden of solitude and isolation was clear even though not often mentioned directly.

### 4.4. Wish to die and the experience of dying in patients with cancer

The cancer patients we interviewed were well informed about their advanced disease. One striking thing about the illness narratives of people with cancer was that many of them deployed similar metaphors in referring to their experience of illness. The metaphor of ‘combat’ was especially frequent: the tumor as the ‘enemy’, the illness against which one must ‘fight’ with all one’s strength. Participants often said that they had fought for a long time, mostly with enormous effort up to the point where they were told or realized that treatment was no longer effective and that the ‘battle was over’. Like people with neurological diseases, cancer patients talked about turning points where dying was (re)considered: at the initial diagnosis, after the first relapse, when writing a living will, or when opting for consistent palliative care. These points were also decisive for the development or reconsideration of a WTD.

Patients reported that they found ‘having cancer’ as particularly socially burdensome. A female patient aged 54, with carcinoma of the upper jaw for 20 years and breast cancer in remission for 10 years, described the moment her second tumor was diagnosed: “I can believe it, yes *[that someone would feel the need to take their own life]*. I came out of the hospital after the diagnosis of cancer and thought, this is what someone who commits suicide feels like.” (P21-I). Another young female patient, 34 years old, with breast cancer (P19-I), spoke about herself in the third person when negotiating cancer, prognosis and dying–as if she were speaking of another person. In the relative’s interview, her sister added: “They [persons with cancer] are people of flesh and blood, with fears, with worries, with uncertainties, up to their neck in catastrophe–so not just the big "C" for "cancer" but also "C" for "catastrophe". […] There are a lot of worse diseases, but just that word [cancer] … If a person hears they have cancer, just that alone is devastating, absolutely devastating! For the families as well.”

Similarly, non-cancer patients referred to cancer as an illness that was even more frightening than their own pathology. A patient with cardiac insufficiency said: “Yes, I think especially, if I were to get cancer. […] That would be a catastrophe for me! […] For some reason I have a horror of that. […] You know, there are some things that one just has a horror of, and you can’t overcome it. […] Yes, yes, cancer of course has a bad reputation.” (P2-II)).

#### Intentions

In cancer patients’ wishes to die we found different intentions, which we have described elsewhere more in detail [7]. They ranged from looking forward to dying but without any intention to hasten it, to a concrete and real desire to act so as to hasten death. Somewhere in between were hypothetical wishes, which contained the condition ‘if … then’. Here, we looked at tendencies in these intentions, which relate to the subjective experience of the cancer trajectory. We observed that hypothetical WTHD, located in a future when something unwanted occurs, played an important role for cancer patients. Many cancer patients we interviewed were members of a Swiss right-to-die organization, even though they often emphasized that it was only to have an option in case things became worse or unbearable: “I signed up for EXIT, purely for safety reasons, in case I wanted to pull the emergency brake. […] I am signed up to EXIT, I sorted that out almost at the same time as the hospice. EXIT would really just be an emergency handle and not because I particularly wanted it. It would be only if I felt I couldn’t get off this train. It wouldn’t be because I actually wanted that.” (P5-I)

More clearly than in other groups, people with cancer seemed to have more imminent ideas about hastening death. Many of them even took actions to accelerate their dying, by making a concrete request for help to hasten death (e.g. to nurses; P20-I), by asking for sedation to hasten death (P12-I), by withdrawing life-sustaining treatment with the idea of accelerating dying (P21-I), by stopping eating with the intent to hasten death (P30-I), by contacting a right-to-die organization and reserving a barbiturate (P13-I; P29-I), or by actively attempting suicide (P32-I).

#### Motivations

As we have described in more detail elsewhere [63], there was a wide variety of reasons, meanings and functions in the WTD of cancer patients, but not necessarily only in relation to their experience of illness. Here, we focus on those aspects that seem characteristic of the experience of being ill and dying of cancer. Many interviewees wished to die because of the high physical and psychological burden of symptoms such as pain, dyspnea, chronic nausea, fatigue, anxiety or depression. They wished to die so that death could put an end to severe suffering, to a situation that was seen as an unreasonable physical or psychological demand, or to end a life they felt was now without value.

Many expressed a WTD at decisive turning points in their illness, as described above: the first diagnosis, the first relapse, and the decision to bring forward palliative care (hospice etc.). For example, this woman of 34, the mother of a toddler, described the moment when the physicians told her that curative treatment for her breast cancer would no longer be helpful:

“You know, I have battled with this stupid cancer for a year and a half. I really tried all the operations, tried all the chemo, and as long as there was even a tiny bit of hope, just the tiniest bit, I still travelled to Lausanne to have an operation, I did everything, but the moment they said, we can’t do any more for you, I said, okay, now it’s finished, now I can’t fight any more, now I don’t even want to fight any more, I would like to die now, I’d like to go now. That was quite clear for me.”

(P17-I)

Some people with cancer told us that their very clear prognosis of dying left them existentially with a lack of perspective, hopeless and speechless, which made them want to die. Others said that the clear certainty of approaching death made it difficult for them to continue to wait until it came naturally; they wished to bring on death so it would happen faster:

“This is no quality of life for me. (…) I just find it a shame that I still have to lie around here for so long. You know, someone else could use this bed. Yes, I mean, I don’t know why this has to be so prolonged.”

(P17-I)

It is notable that this young patient also had a strong wish to live, which she explained in terms of her hope of cherishing good moments with her family and with her self-perceived role as a mother, daughter and spouse.

Several patients explained their WTD as due to physical suffering and the lack of any prospect of getting better. A woman of 64, with ovarian cancer, told us one week after attempting suicide by taking benzodiazepines:

“I’ve been really unwell in the last couple of months. And yes, sometimes a good day, but then bad, bad, bad. You know, if you feel ill for days, and you can’t eat anything, if you’re almost throwing up when you just smell food, then I thought, there’s no value in this any more. I just want to go to sleep. […] I just thought, I can’t go on any more. And the feeling sick doesn’t get any better […] every day is just torture, isn’t it?”

(P32-I)

As in other patient groups, the feeling of being a burden to others often underpinned a WTD. Interviewees talked about the many different types of burden they believed they had inflicted on others: time-consuming physical care, emotional distress (sorrow, fatigue, worry), but also financial pressures, or illness-related nuisances such as foul odors, stoma or tubes. One lady with an exacerbating breast cancer told us about her WTHD:

“Yes, well, I thought umpteen times, I’d like to have someone from EXIT come, so I signed up, with Dignitas as well, um, because I thought: yes, well, if it becomes so unbearable that, that everyone around me has to hold their nose. That was the worst, I think, then I wanted to, well, break off the exercise.”

(P4-I)

Some hoped, therefore, that their death would free others from their burden. Others told us they experienced a WTD because of feelings of shame (of being dependent, of having a distorted body, of being incontinent) and of being exposed to what A.W. Frank called the “will of the body” [66], meaning the arbitrariness of illness with uncontrollable consequences for the person’s life.

For many patients, autonomy and self-determination were important issues. The WTD was sometimes understood as an act of regaining agency, by determining when it was enough or the moment of one’s own death. But among the meanings attributed by patients to their WTD, autonomy was only one among many others and often not the primary motivation.

#### Social interactions

Cancer patients were dependent on support for shorter times and often less heavily than the three other patient groups in the final phase of life. The perception of being a burden to others–felt not only about families or healthcare providers, but also sometimes in terms of society in general–was frequently expressed by our interviewees in this group. One frail elderly cancer patient, who had no family members and whose only caregiver was a neighbour, expressed a persistent WTD to the hospice physician during the morning rounds. This man, who had dedicated his entire life to the military as a high-ranking general, believed he did not deserve the costs of care, that he was a burden to society and he therefore wanted to die. The wish to die vanished under palliative care in a hospice. What persisted was the wish for control in the next crisis, but the patient then agreed to use a palliative approach including deep sedation. This case is discussed in detail by Gudat [67].

While many patients reported in the interviews that they had deepened communication with and understanding of their families and friends during or because of illness, others told us that their relationships had broken down upon diagnosis or as the illness advanced, or that they had been abandoned by their families or friends because of their illness. One woman of 54 (P5-I), with breast cancer, narrated how at the time of diagnosis her partner and her brother turned their backs on her:

“Well, it started with my partner just disappearing after three weeks, and plunging into a marriage to another woman and nine months later becoming a father. And I had wanted children all my life long.”

“Well, my middle brother, for example, didn’t speak to me for a year and avoided contact completely, so that he didn’t have to come into contact with me. […] And when that [*the cancer diagnosis*] came, he hung up the phone and that was it. [*laughs briefly*] Well, that’s, from my point of view that’s really him being overwhelmed.”

(P5-I).

## 5. Discussion

In our study of people undergoing the same illness or frailty due to very old age, we found recurring themes and experiences that were used by the interviewees to explain the emergence of their WTD, as well as differences between the groups that characterized the WTD of the different trajectories.

### 5.1. Typical patterns, differences and similarities in wishes to die and subjective experiences of dying

#### Differences and typical patterns

Many of the aspects that make up a particular WTD are of course bound to the very personal experiences, moral values and preferences within which this wish is situated [3] [4]. Our study showed that for WTD there are recognizable, typical illness-related features or patterns that are common to the subjective experiences of patients with the same illness and dying trajectory, but which show differences when compared to other dying trajectories. Being aware of these aspects might facilitate communication about and the exploration of WTD with individual patients, but also gives important insights into the particular challenges of each of these dying trajectories overall [13] [14].

Patients with neurological diseases who expressed a WTD were often concerned with how dying would happen and how they could cope with the physical decline they knew would come. They feared physical suffocation, dependence on others and on breathing support, and being rendered immobile. Similar results have also been reported by other studies [15] [29] [40]. These fears are the reason why many of the patients expressed a hypothetical WTHD for the future, should their condition become unbearable, while others in this group also expressed current WTD with and without the wish to hasten death. Hypothetical WTD indicated a need for security, and the fear of complete dependence and loss of sovereignty. The people we interviewed were well informed and clear about the fact that in the near future, decisions about life-sustaining measures (artificial nutrition and hydration, respirator), and about the type and amount of support they knew they could count on, would need to be taken. For ALS patients in particular, it was a relief and counteraction to a WTD to make an advance care plan together with their physicians, covering possible treatment options over the course of time, including options for future palliative sedation in case of refractory breathlessness, as well as defining critical points that would require more communication and shared decision making. Patients reported how important it was for them to be reassured of receiving palliative sedation in a possible future terminal situation and that they would not have to suffocate, and how this reassurance counteracted a WTD. We are aware that this constellation was dependent on the standard procedure elaborated for advance care planning in the ALS center where we recruited interviewees, and that this might differ in other settings or countries.

Persons with organ failure stated their WTD in the context of an oscillation between life threatening health crisis and re-stabilization. Their WTD often reflected this: all of them had expressed a WTHD during a life-threatening crisis and many of them had hypothetical WTD for the future, should there be another crisis. These hypothetical WTD could be understood as a means of control that protected them from further crises of unwanted experiences and pain. While there are several studies reporting the suffering and concerns of people living with organ failure [53], there are studies that investigate the WTD in this illness group and see this constellation as a reason for a WTD [15].

We were also able to show very clearly that the strain of engaging with life again after a life-threating health crisis, the feeling of being stuck in an in-between state, the unpredictability and great complexity of illness, could all be a motivation for a WTD. The continual repetition of thinking they were going to die, and then having to start again and find their way back into life, was described by these patients as demanding enormous amounts of energy. Some wanted to die because they felt the effort to be too much. Others said they wished to die because they did not want to feel stuck in an “intermediate state” in which they were not dying, but not living well either. Finally, one patient expressed a wish to die because of the fact that their pathologies finally became so complex that treatments were mutually exclusive. To our knowledge there are no similar in-depth studies on the experiences and needs of patients with organ failure and a WTD, and further investigations are needed.

Older, frail persons in our study situated their WTD as being a result of their inner and outer reality becoming increasingly complex. They felt overwhelmed by that. Feelings of alienation from society and other generations, physical deterioration, increasing dependence, institutionalization, and the loss of friends and relatives produced a feeling of estrangement and a reduced life world. These are subjective motivations that are not described as such in other studies, but might well be connected to or masked by the more objective factors used in quantitative studies. As described in the introduction, known objective factors for a WTD in frail elderly people include not being in a partnership, greater frequency of depressive symptoms, loss of autonomy, financial problems, limited social network, urinary incontinence, a negative perception of one’s own physical health, polymorbidity, higher levels of stress, sleep problems, and living in a nursing home [30][45][46][47][48][50]. Our study indicated that even more important factors were perceptions of a change in thought processes (slower thinking, forgetfulness, repetitive thoughts), and general loss of interest in life. Social isolation and loneliness were less frequently mentioned by our interviewees as reasons for a WTD, but might nevertheless have played an important role as indicated by other studies [9][30][49]. We can only hypothesize that for elderly persons, this is a difficult topic to talk about, which makes it an aspect to be aware of in assessing a WTD in frail elderly people.

People with cancer expressed a WTD in situations of high physical burden and emotional suffering [15]. They wanted to let death put an end to severe suffering, to end a situation that they saw as unreasonably demanding in either a physical or psychological sense, or to end a life they felt was now without value [63]. Cancer patients were mostly well informed about their prognosis and their approaching death. Turning points in the illness trajectory (diagnosis, relapse, move into palliative care) triggered reflections on dying and sometimes also a WTD. Lack of a longer-term perspective and hopelessness due to the clear awareness of approaching death were frequently mentioned by the interviewees as reasons for their WTD. This seems to be particularly true for cancer patients (perhaps like patients with neurological diseases) and less so for people with organ failure, who focus on overcoming the next crisis, or by the frail elderly, who have more diffuse pathologies and dying trajectories.

#### Similarities

Hypothetical WTD were expressed by all patient groups, but were differently situated and diversely distributed along the different trajectories. As those with neurological diseases or cancer were often very clear about their prognosis and the proximity of dying, hypothetical WTD were expressed in connection with ideas about concrete situations or circumstances they knew might occur, and which they felt would be intolerable or unbearable. Patients with organ failure who had experienced a life-threatening crisis expressed hypothetical WTD for the next crisis, in order to avoid going through it. For people with neurological diseases and cancer, these hypothetical WTD seemed to serve the function of maintaining a sense of agency in advance of the dying process, while for those with organ failure, they were a preventive act to avoid having to go through another unbearable crisis. In all pathologies, we noticed that the hypothetical WTD of patients who considered themselves close to dying were formulated in a way that felt emotionally closer or more realistic than the hypothetical WTD of patients who thought they were still quite far from death. In the latter, hypothetical WTD were formulated more abstractly, and for the moment seemed to require a dialogue on advance care planning about rigorous symptom management. We are not currently aware of any study investigating hypothetical WTD in different disease trajectories. Further research on this could give deeper insights and would enable communication and assistance to be more personalized. In assessing these hypothetical WTD, it is important to evaluate how patients see themselves in the progress of their illness and how far they consider themselves to be from dying. However, for illnesses such as ALS, MS or cancer, time until and circumstances of death were more foreseeable than for patients with organ failure or frailty due to age.

Particular moments that triggered WTD: In all patient groups except the frail elderly who have a less predictable dying trajectory, we were able to identify clear moments that triggered WTD. For neurological patients, it was when they had to consider starting tube feeding or respirator support. The onset of advanced breathlessness, spasmodic contractions or immobilization that significantly compromised quality of life and indicated the proximity of death, and moments when deciding to initiate palliative sedation, were other important timepoints for neurological patients. Patients with organ failure experienced time-restricted WTHD during an acute crisis and for future crises. Patients with cancer often reflected on a WTD soon after the initial diagnosis, the first relapse, significant aggravations, and during the shift from curative into palliative care (if understood as a clear ‘cut’ in patient-physician communication: “there is nothing we can do”; “then I stopped fighting”). We can hypothesize that, for frail elderly people, moments such as giving up one’s home and moving into care facilities, and losses of important relationships or physical abilities might be similar timepoints that trigger a WTD. In the healthcare of all pathologies these critical moments deserve particular attention, and increased communication in the process of care, planning and decision making. Further research could investigate these particular points that trigger a WTD in the respective disease and dying trajectories more in detail.

Feeling of being a burden to others: Patients in all groups said they suffered because of being dependent and feeling that they were a burden to others, although it was a less important theme in persons with organ failure. Many gave this as a major motivation for their WTD. This is also known from other studies [40]. The sense of being a burden was most strongly stated by cancer patients (including the fear of being an emotional burden on their relatives) and frail elderly persons. Frail people in particular felt not only that they were a burden to their relatives and health professionals, but often also to society or the younger generation as a whole. Feelings of not contributing meaningfully to the lives of others, and having to be cared for instead of being carers themselves as they had been accustomed, were associated with elderly people’s WTD. Living in a meaningful partnership seemed to act as a protection against WTD. The self-perception of being a burden was mentioned by patients with neurological diseases, as has been found by other studies [7, 29]. In a longitudinal quantitative interview study with 93 ALS patients, Dorothée Lulé and colleagues [29] found the ‘feeling of being a burden to others’ “a significant determinant of decision against life-prolonging treatment and for the desire to hasten death”, which seemed to be even more important in ALS patients on ventilation and/or PEG. In our study, neurological patients nevertheless spoke most positively about relationships with their relatives (with some exceptions). While their relatives tended to be positive, the relatives also problematized the objective burden on themselves of providing long-term care. In patients with organ failure this theme was mentioned less frequently, which is noteworthy since care for persons with organ failure is often associated with a significant burden on relatives. For patients with a WTD across all patient groups, the theme of being a burden on relatives deserves particular attention in the healthcare context [63]. It might be culturally and gender-dependent and should be investigated more thoroughly. (For the similarities and differences of WTD between the four dying trajectories see Tables 3 and 4).

**Table 3:** Similarities of wishes to die in all four trajectories.

Similarities of wishes to die in all four trajectories (neuro, organ failure, frailty, cancer)
WTD are complex and dynamic; WTD appear frequently in the experience of dying; subjective meaning of WTD includes a) intentions, b) motivations, c) social interactions; a variety of intentions of WTD are present in all patient groups; WTD can be fluctuant and change over time, even close to dying; WTD can contain various wishes next to each other: wishes to live, various wishes to die with or without wishes to hasten death; hypothetical WTD expressed in all groups; hypothetical WTD of persons who think themselves close to dying are more realistic and concrete than those who think themselves far from dying; in all groups there are particular moments or incidences that trigger a WTD; feelings of ‘being a burden to others’ appear as reason for WTD in all trajectories.

**Table 4:** Differences of wishes to die among the four trajectories.

Differences and typical patterns of wishes to die among the four trajectories	
Neuro	Hypothetical WTD: should conditions in future become unbearable. Fears behind hypothetical WTD: physical suffocation, dependence on others and on breathing support, being rendered immobile, fear of being completely dependent and losing sovereignty. For some, hypothetical WTD served the function of being in control when confronted with increasing loss of autonomy. Points in the illness trajectory that triggered a WTD: considering starting tube feeding or respirator support, the onset of advanced breathlessness, spasmodic contractions or immobilization, significantly compromised quality of life and deciding to initiate palliative sedation. Advanced care planning can counteract a WTD.
Organ failure	Wish to hasten death can appear during a life-threatening crisis. Hypothetical WTD: should there be another life-threatening crisis, to avoid unwanted experiences and pain. Motivations for a WTD: significant deterioration of health, fear of the next life-threatening crisis, feeling of being stuck in a state between life and death, too much effort of starting life after crisis, the unpredictability and great complexity of illness. Points in the illness trajectory that triggered a WTD: during an acute crisis.
Frailty	Approaching death is not an abstract future event, but something to relate to and ponder about. Repetitively expressed WTD are often specific to this patient group. Motivations for WTD: Feelings of being worthless, of alienation from society and other generations, physical deterioration, loss of activity, increasing dependence, institutionalization, the loss of friends and relatives, feeling of estrangement, perceptions of a change in thought processes, general loss of interest in life, social isolation and loneliness besides more objective factors described by other studies. Together with cancer patients, fear of being a burden to others strongest in this patient group. Points in the illness trajectory that triggered a WTD: giving up one’s home, moving into care facilities, loss of important relationships or physical abilities.
Cancer	WTD often expressed in situations of high physical burden and emotional suffering. Frequent hypothetical WTD for the future. Motivations for WTD: to let death put an end to severe suffering, to end a situation perceived as unreasonably demanding, or to end a life they felt was now without value, wanting to control of future events, lack of a longer-term perspective and hopelessness due to the clear awareness of approaching death. Together with frail elders, fear of being a burden to others strongest in this patient group. Points in the illness trajectory that triggered a WTD: diagnosis, relapse or diagnosis of metastasis, significant aggravations or during the shift from curative into palliative care.

### 5.2. Conclusions and practical implications

The subjective experience of dying trajectories provides only a partial explanation for the emergence and nature of WTD. WTD are always subjective and need to be understood in terms of a variety of individual factors, such as personal values, moral understandings, life history, and social interactions [3, 4]. The experience of one’s own illness when approaching death, however, forms the essential background against which people negotiate their life’s end and may eventually express a WTD. We were able to show that, in the narratives of persons with a WTD and with similar dying trajectories, there are recurring considerations connected to the particular illness experiences, which combine with other, more personal concerns to explain why a WTD is present. We noticed that the body of research in this field is small and does not permit a detailed comparison with our findings.

The strengths of our study are: (a) its prospective approach, which allows a detailed analysis of first-person accounts and provides insight into the personal experience and subjective evaluation of a WTD. (b) The results give an empirically grounded contribution to the conceptualization of WTD statements in different dying trajectories. This is the first study to investigate WTD in detail in non-cancer patients, and provides comparative findings on cancer and non-cancer patients. (c) The study is a qualitative one with a relatively large number of interviewees (N = 62 patients). (d) As we interviewed people on average 53 days before death in the second study (non-cancer; NRP76) and 23 days in the first study (cancer; Oncosuisse), the data report on WTD statements in a situation of relative proximity to dying.

However, our study also has limitations: (1) The sample was limited to patients with access to palliative care and hospice care. We did not interview patients who were not perceived to be in a palliative situation, those not under palliative care, nor persons who exclusively decided to hasten death through one of the right-to-die organizations. Such studies still need to be done. (2) The inclusion of patients in the study depended on physicians’ judgment. This criterion may have caused a certain selection bias, but was justified by the need to protect particularly vulnerable patients from burdening themselves by volunteering for the study; it is part of good practice in palliative care research.

Illness-related considerations alone do not give a comprehensive insight into the entirety of the WTD, but provide important information on a) the challenges that particular patient groups commonly face and that might lead to the formulation of a WTD; and b) what to investigate when wanting to understand the WTD of a particular patient. Looking at the WTD through a ‘trajectory lens’ shows that people dealing with similar trajectories are often confronted with similar questions and concerns due to facing common challenges [13–15]. These challenges set the conditions under which WTD are articulated.

A deeper understanding of the concerns and challenges and the particular setting of an illness and dying trajectory in which a WTD arises can facilitate communication about end-of-life wishes and be helpful in assessing particular patients’ needs and fears more accurately and responsively [13]. This requires WTD to be actively addressed as part of routine palliative care, ideally before a wish to hasten death has developed, bearing in mind the context and characteristics of experience that can come with living through a particular illness trajectory. The awareness of these characteristics, such as critical points in the trajectory that might trigger a WTD, of typical social issues such as feeling that one is a burden to others, or of particular needs to plan ahead and work out how to arrange the most dignified possible way of dying, is vital to improving care for palliative patients negotiating a WTD.

## Supporting information

S1 Supporting information Patient information sheet German. (PDF)

S2 Supporting information Patient information sheet English. (PDF)

S3 Supporting information Patient informed consent form German. (PDF)

S4 Supporting information Patient informed consent form English. (PDF)

S5 Supporting information Original German quotes in relevant interview context. (PDF)
