# Supplementary material for: Wishes to die at the end of life and subjective experience of four different typical dying trajectories. A qualitative interview study
Source: PLoS One. 2019 Jan 17;14(1):e0210784. doi: 10.1371/journal.pone.0210784 (PMC6336242; doi:10.1371/journal.pone.0210784)
Supplement: S1 Supporting information — (PDF) [file pone.0210784.s001.pdf]

Gedanken von schwerkranken Menschen zu ihrer Lebenssituation und zu ihrem Leben und Sterben

Nationalfonds-Projekt 406740 145089

Studienleitung
Dr. med. Heike Gudat, HOSPIZ IM PARK, Klinik für Palliative Care, Arlesheim
Prof. Christoph Rehmann-Sutter, Institut für Medizingeschichte und Wissenschaftsforschung IMWGF, Universität zu Lübeck, D-23552 Lübeck

Interviewteam
Kathrin Ohnsorge (Leitung), Heidi Gass, Lucia Stäubli, Nina Streeck

INFORMATION FÜR DIE TEILNEHMENDEN PATIENTINNEN UND PATIENTEN

Sehr geehrte Dame, sehr geehrter Herr

Wir laden Sie ein, an unserem Forschungsprojekt teilzunehmen. Wir möchten untersuchen und verstehen, wie schwerkranke und alte Menschen zu ihrem Leben und ihrer Endlichkeit stehen. Gerne möchten wir mit Ihnen über Ihre Gedanken, Wünsche und über Ihre persönlichen Einstellungen zum Leben und zum Sterben sprechen.
Ihre Auskünfte, die wir selbstverständlich vertraulich behandeln und nur in anonymer Form verwenden werden, wären ausserordentlich wertvoll, um die Situation kranker und alter Menschen genauer zu verstehen und sie besser unterstützen zu können. Die Teilnahme an dieser Interview-Studie ist für Sie vollkommen freiwillig.

Hintergrund für diese Untersuchung

Nur wenige Studien haben sich bisher mit der Frage befasst, was schwerkranke und alte Menschen beschäftigt und wie sie über das Leben und das Sterben denken.

Stollenrain 12 · CH 4144 Arlesheim
Tel +41 (0)61 706 92 22 · Fax +41 (0)61 706 92 20
www.hospizimpark.ch · info@hospizimpark.ch

Die meisten Untersuchungen stützen sich auf die Beobachtungen der behandelnden Ärzte und Pflegenden. Uns scheint es jedoch wichtig, die Gedanken und Perspektiven der direkt betroffenen Menschen zu untersuchen.

Vorgehensweise

Der wichtigste Teil unserer Arbeit besteht darin, die Betroffenen, Angehörigen und Betreuenden zu interviewen. Wir würden gerne mit Ihnen ein bis höchstens drei Interviews durchführen. Die Teilnahme am Interview und die Beantwortung der Fragen sind zu jedem Moment vollständig freiwillig. Das Gespräch wird von zwei Mitgliedern der Forschungsgruppe geführt und soll höchstens eine Stunde dauern.

Wir möchten im Gespräch von Ihnen erfahren, welche Erfahrungen Sie während Ihrer Erkrankung gemacht haben und welche Gedanken über Ihre Situation und über die Zukunft Ihnen durch den Kopf gehen. Es geht uns ausschliesslich um Ihre persönliche Sicht der Dinge. Auf keinen Fall sollen Ihre Einstellung oder Ihre Entscheidungen moralisch bewertet werden.

Die Interviews werden zu einem mit Ihnen vereinbarten Zeitpunkt und am Ort Ihrer Wahl geführt. Die Gespräche werden auf Tonband aufgenommen und getippt. Sie dürfen das Manuskript jederzeit einsehen und Korrekturen oder Kommentare anbringen.

Während Ihrer Teilnahme an der Studie werden die wichtigsten medizinischen Ereignisse auf einem Verlaufsblatt durch das Behandlungsteam festgehalten. Dieses Verlaufsblatt gehört zur Routinedokumentation Ihrer Behandelnden und wird unabhängig von dieser Studie ausgefüllt.

Auch diese Dokumente sind für Sie jederzeit einsehbar. Die teilnehmenden Institutionen arbeiten vollkommen unabhängig. Die Studie wird vom Schweizerischen Nationalfonds und der Gottfried und Julia Bangerter-Rhyner-Stiftung unterstützt.

Befragung der Angehörigen

Neben den Interviews mit Ihnen würden wir gerne, wenn es möglich ist, auch ein Interview mit Ihren Angehörigen oder Ihnen nahestehenden Menschen durchführen. Dieses Interview dient uns dazu, weitere Informationen zusammeln, um Ihre Situation besser zu verstehen. Wir werden dieses zusätzliche Interview aber nur durchführen, wenn Sie damit einverstanden sind. Gegenüber Ihrem Angehörigen werden in jedem Fall keine Aussagen erwähnt, die Sie in Ihrem eigenen Interview gemacht haben.

Stollenrain 12 · CH 4144 Arlesheim
Tel +41 (0)61 706 92 22 · Fax +41 (0)61 706 92 20
www.hospizimpark.ch · info@hospizimpark.ch

Interview mit einem betreuenden Arzt und einer Pflegefachperson
Um zu verstehen, wie Ihre Situation vom Behandlungsteam wahrgenommen wird, ist es wichtig, auch ein Interview mit einem Arzt und einer Pflegefachperson zu führen, die Sie betreuen. Die Interviews werden unabhängig voneinander geführt. Auch gegenüber dem Arzt und der Pflegefachperson werden keine Aussagen erwähnt, die Sie in Ihrem eigenen Interview gemacht haben.

Datenschutz

Alle Mitglieder des Forschungsteams unterstehen dem Patientengeheimnis. Alle persönlichen Daten werden vertraulich behandelt, d.h. die Bänder und Protokolle werden nicht an Dritte weitergegeben und die Informationen werden vor der weiteren Verarbeitung anonymisiert. Ihre persönlichen Daten dürfen ohne Ihren ausdrücklichen Wunsch für keinen anderen Zweck verwendet oder an Dritte preisgegeben werden, auch nicht an Ihre Angehörigen oder an Ihre behandelnden Ärzte oder andere Therapeuten. Ihr Name wird in keiner Weise in Rapporten oder Publikationen, die aus der Studie hervorgehen, veröffentlicht. Die anonymisierten Daten dieser Studie werden nach deren Beendigung an einem sicheren Ort aufbewahrt. Die Tonbandaufnahmen der Interviews werden nach Studienende vernichtet.

Freiwilligkeit und Rückzugsrecht

Ihre Teilnahme an dieser Studie ist freiwillig. Wenn Sie nicht an dieser Studie teilnehmen wollen, haben Sie keine Nachteile für Ihre medizinische Betreuung zu erwarten. Das gleiche gilt, wenn Sie Ihre einmal gegebene Einwilligung zu einem späteren Zeitpunkt widerrufen. Diese Möglichkeit haben Sie jederzeit. Einen allfälligen Widerruf Ihrer Einwilligung bzw. den Rücktritt von der Studie müssen Sie nicht begründen. Im Fall eines Widerrufs werden die bis zu diesem Zeitpunkt erhobenen Daten weiter verwendet, sofern Sie damit einverstanden sind.

Entschädigung

Für die Teilnahme an dieser klinischen Studie erhalten Sie keine Entschädigung.

Kontakt
Dr. med. Heike Gudat Keller
HOSPIZ IM PARK, Klinik für Palliative Care
Stollenrain 12, 4144 Arlesheim

Telefon: 061 706 92 22
e-mail: heike.gudat@hospizimpark.ch
