# Supplementary material for: Wishes to die at the end of life and subjective experience of four different typical dying trajectories. A qualitative interview study
Source: PLoS One. 2019 Jan 17;14(1):e0210784. doi: 10.1371/journal.pone.0210784 (PMC6336242; doi:10.1371/journal.pone.0210784)
Supplement: S2 Supporting information — (PDF) [file pone.0210784.s002.pdf]

Attitudes of seriously ill people towards their situation, living and dying

Swiss National Science Foundation Project 406740 145089

Principal investigators
Dr. med. Heike Gudat, HOSPIZ IM PARK, Klinik für Palliative Care, Arlesheim
Prof. Christoph Rehmann-Sutter, Institut für Medizingeschichte und Wissenschaftsforschung IMWGF, Universität zu Lübeck, D-23552 Lübeck

Interview team
Kathrin Ohnsorge (head), Heidi Gass, Lucia Stäubli, Nina Streeck

INFORMATION FOR PATIENTS TAKING PART IN THE STUDY

Dear participant,

We invite you to take part in our research project. We would like to investigate the way that seriously ill and older people think about their life and its finitude. To understand this better we would like to talk with you about your thoughts, wishes and personal attitudes towards life and death.
Your information, which will be kept confidential and used only in anonymised form, would be extraordinarily valuable in understanding better the situation of ill and older people and in being better able to support them.
Participation in this interview study is completely voluntary.

Background to this study

Only a few studies have so far investigated the concerns of seriously ill and older people and how they think about living and dying.

Stollenrain 12 · CH 4144 Arlesheim
Tel +41 (0)61 706 92 22 · Fax +41 (0)61 706 92 20
www.hospizimpark.ch · info@hospizimpark.ch

Most of the existing studies are based on observations by doctors and nurses who are treating them. However, we think it is important to understand the thoughts and perspectives of the people who are most directly affected.

Procedure

The major part of our study involves interviewing seriously ill and older people, their families and carers. We would like to carry out up to three interviews with them. Participation in the interview is completely voluntary, and you do not have to answer any questions you prefer not to. The conversation will be carried out by two members of the research group and should last no more than one hour.

In conversation with you we would like to learn about the experiences you have had during your illness, and what thoughts are in your mind about your situation now and in the future. We are only interested in your personal view of things, and will not make any judgements about attitude or your decisions.

The interviews will be carried out at a time agreed with you and at a place of your choosing. The conversations will be recorded on tape and then transcribed. You will be able to see the transcription at any time and make corrections or comments.

During your participation in the study, any significant medical events will be recorded by your medical team. This record is part of the routine documentation of your medical care, and is completely independent of this study. You may also see these documents at any time.

The participating institutions are working completely independently. The study is funded by the Swiss National Science Foundation and the Gottfried und Julia Bangerter-Rhyner Stiftung.

Family interviews

In addition to the interviews with you, we would also like to interview your family members or other people who are close to you. This interview will give us further information so that we can understand your overall situation better. We will however only carry out this interview with your consent. In no case will we mention to your family anything you have said in your own interview.

Stollenrain 12 · CH 4144 Arlesheim
Tel +41 (0)61 706 92 22 · Fax +41 (0)61 706 92 20
www.hospizimpark.ch · info@hospizimpark.ch

Interview with a treating physician and a member of the nursing staff
To understand how your medical team perceive your situation, it is important that we also interview a doctor and a nurse who are treating you. The interviews will be carried out independently of each other. We will not mention to the doctor or nurse anything you have said in your own interview.

Data protection

All members of the research team are subject to regulations governing patient confidentiality. This means that all personal details will be handled in confidence, i.e. the recordings and transcripts will not be passed on to third parties, and all information will be anonymised before processing it any further. Your personal data will not be used for any purpose other than this research or disclosed to third parties, not even your family members or your treating physicians or other healthcare professionals, without your express request. Your name will not be disclosed in any reports or publications resulting from this study. The anonymised data collected will be stored in a safe place after the study has ended. The tape recordings of the interviews will be destroyed after the study has ended.

Voluntary participation and withdrawal

Your participation in this study is entirely voluntary. If you decide not to take part in this study, it will not affect your medical treatment in any way. The same applies should you agree to participate but change your mind at a later stage. You have the right to withdraw from the study at any point, without needing to give a reason. If you withdraw from the study the data collected up to this point will continue to be used, as long as you agree to this.

Financial compensation

You will not receive any payment for taking part in this clinical study.

Contact
Dr. med. Heike Gudat Keller
HOSPIZ IM PARK, Klinik für Palliative Care
Stollenrain 12, 4144 Arlesheim, Switzerland

Telefon: 061 706 92 22
e-mail: heike.gudat@hospizimpark.ch
