# Supplementary material for: Wishes to die at the end of life and subjective experience of four different typical dying trajectories. A qualitative interview study
Source: PLoS One. 2019 Jan 17;14(1):e0210784. doi: 10.1371/journal.pone.0210784 (PMC6336242; doi:10.1371/journal.pone.0210784)
Supplement: S3 Supporting information — (PDF) [file pone.0210784.s003.pdf]

HOSPIZ IM PARK
Klinik für Palliative Care

Schriftliche Einverständniserklärung zur Teilnahme an einer klinischen Studie für Patientinnen und Patienten

Bitte lesen Sie dieses Formular sorgfältig durch.
Bitte fragen Sie, wenn Sie etwas nicht verstehen können oder etwas wissen möchten.

Titel der Studie	**Gedanken von schwerkranken Menschen zu ihrer Lebenssituation und zu ihrem Leben und Sterben**
Studienleiter/in:	Dr. med. Heike Gudat Keller und Prof. Christoph Rehmann-Sutter

Ort des Interviews:

Interviewerteam:

Interviewte Person

Name und Vorname:

Geburtsdatum:

Ich wurde von der unterzeichnenden Ärztin bzw. von der interviewenden Person mündlich und schriftlich über die Ziele und den Ablauf der Studie informiert.

Ich habe die schriftliche Patienteninformation vom 01.12.2013 zur oben genannten Studie gelesen und verstanden. Meine Fragen in Zusammenhang mit der Teilnahme an dieser Studie sind mir zufriedenstellend beantwortet worden. Ich kann die schriftliche Patienteninformation behalten und erhalte eine Kopie dieser schriftlichen Einverständniserklärung.

Ich hatte genügend Zeit, um meine Entscheidung zu treffen.

Stollenrain 12 · CH 4144 Arlesheim
Tel +41 (0)61 706 92 22 · Fax +41 (0)61 706 92 20
www.hospizimpark.ch · info@hospizimpark.ch

Ich bin einverstanden, dass die zuständigen Fachleute des Studienteams in meine Originaldaten Einsicht nehmen dürfen, jedoch unter strikter Einhaltung der Vertraulichkeit.

❐ Ja, ich bin einverstanden, dass ein Interview mit meinem/r Angehörigen oder dem nahe stehenden Menschen durchgeführt wird. Ich möchte, dass folgende Person interviewt wird, sofern sie damit einverstanden ist: .... Name der Person).

❐ Nein, ich bin nicht einverstanden, dass ein Interview mit einem/r Angehörigen oder einem nahe stehenden Menschen durchgeführt wird.

❐ Ja, ich bin einverstanden, dass das Studienteam eine Pflegefachperson und einen Arzt interviewen, die mich betreuen. Ich entbinde die interviewte Person von der Schweigepflicht.

❐ Nein, ich bin nicht einverstanden, dass oben genannte Fachpersonen interviewt werden.

Ich bin darüber informiert, dass eine Versicherung Schäden im Rahmen der Studie deckt.

Ich bin einverstanden, dass der Hausarzt über die Studienteilnahme informiert wird.

Ich weiss, dass meine persönlichen Daten nur in anonymisierter Form zu Forschungszwecken weitergegeben werden. Ich bin einverstanden, dass die zuständigen Fachleute des Studienauftraggebers, der Behörden und der Kantonalen Ethikkommission zu Prüf- und Kontrollzwecken in meine Originaldaten Einsicht nehmen dürfen, jedoch unter strikter Einhaltung der Vertraulichkeit.

Ich nehme an dieser Studie freiwillig teil. Ich kann jederzeit und ohne Angabe von Gründen meine Zustimmung zur Teilnahme widerrufen, ohne dass mir deswegen Nachteile bei der weiteren medizinischen Betreuung entstehen.

Im Interesse meiner Gesundheit kann mich die Prüfärztin jederzeit von der Studie ausschliessen.

Ort, Datum	Unterschrift der Teilnehmerin/des Teilnehmers

Bestätigung der Studienleiterin bzw. des Interviewers / der Interviewerin
Hiermit bestätige ich, dass ich dem/der Patient/in Wesen, Bedeutung und Tragweite der Studie erläutert habe. Ich versichere, alle im Zusammenhang mit dieser Studie stehenden Verpflichtungen zu erfüllen. Sollte ich zu irgendeinem Zeitpunkt während der Durchführung der Studie von Aspekten erfahren, welche seine/ihre Bereitschaft zur Teilnahme an der Studie beeinflussen könnten, werde ich ihn/sie umgehend darüber informieren.

Ort, Datum	Unterschrift der Studienleiterin bzw. des Interviewers/der Interviewerin

Nationalfonds-Projekt 406740 145089.
Informed Consent **Patient.** Version 02 vom 01.12.2013

Stollenrain 12 · CH 4144 Arlesheim
Tel +41 (0)61 706 92 22 · Fax +41 (0)61 706 92 20
www.hospizimpark.ch · info@hospizimpark.ch
