# Supplementary material for: Wishes to die at the end of life and subjective experience of four different typical dying trajectories. A qualitative interview study
Source: PLoS One. 2019 Jan 17;14(1):e0210784. doi: 10.1371/journal.pone.0210784 (PMC6336242; doi:10.1371/journal.pone.0210784)
Supplement: S4 Supporting information — (PDF) [file pone.0210784.s004.pdf]

HOSPIZ IM PARK
Klinik für Palliative Care

Written voluntary informed consent for patients to participate in a clinical study

Please read this form through carefully.
Please ask if you do not understand anything, or would like further information.

Study title **Gedanken von schwerkranken Menschen zu ihrer Lebenssituation und zu ihrem Leben und Sterben (Attitudes of seriously ill people towards their situation, living and dying)**

Principal investigators: Dr. med. Heike Gudat Keller and Prof. Christoph Rehmann-Sutter

Interview location:

Interviewer team:

Interviewee

Surname, first name:

Date of birth:

I have been informed, verbally and in writing, about the study's objectives and procedure by the physician whose signature is given below and/or by the named interviewer.

I have read and understood the written Patient Information dated 01.12.2013 on the study. Any questions I have about participation in this study have been answered to my satisfaction. I can keep the written Patient Information and will receive a copy of this written declaration of voluntary informed consent to participate.

I have been given enough time to make my decision.

Stollenrain 12 · CH 4144 Arlesheim
Tel +41 (0)61 706 92 22 · Fax +41 (0)61 706 92 20
www.hospizimpark.ch · info@hospizimpark.ch

I consent to the members of the research team consulting my original data, but only with strict observation of confidentiality

❒ Yes, I consent to an interview being carried out with a member of my family or other person close to me. I would like the following person to be interviewed, provided they consent: .... Name of person).

❒ No, I do not consent to an interview being carried out with a member of my family or other person close to me.

❒ Yes, I consent to the study team interviewing a nurse and a doctor from my medical team. I release the medical interviewees from patient confidentiality.

❒ No, I do not consent to an interview being carried out with healthcare professionals from my medical team.

I have been informed that there is insurance cover for any harm that might occur as part of the study.

I consent to my GP being informed about my participation in the study.

I understand that my personal data will only be passed on in anonymised form for research purposes. I give my consent for the relevant experts from the study sponsor, the authorities, and the Cantonal Ethics Committee to have access to my original data for monitoring purposes, but only with strict observation of confidentiality.

I am taking part in this study of my own free will. I understand that I can withdraw my consent to participate at any time and without giving reasons, and without this having any effect on my medical care.

In the interests of my health, the investigating physician may exclude me from the study at any time.

Place, date	Signature of participant

Confirmation by the principal investigator and/or interviewer
I confirm that I have explained the study's nature, significance and scope to the patient. I confirm that I will fulfil all my obligations in relation to this study. If at any time during the study any information emerges that might influence the patient's willingness to participate in the study I will inform him/her immediately.

Place, date	Signature of principal investigator and/or interviewer

Stollenrain 12 · CH 4144 Arlesheim
Tel +41 (0)61 706 92 22 · Fax +41 (0)61 706 92 20
www.hospizimpark.ch · info@hospizimpark.ch
