# Supplementary material for: Wishes to die at the end of life and subjective experience of four different typical dying trajectories. A qualitative interview study
Source: PLoS One. 2019 Jan 17;14(1):e0210784. doi: 10.1371/journal.pone.0210784 (PMC6336242; doi:10.1371/journal.pone.0210784)
Supplement: S5 Supporting information — (PDF) [file pone.0210784.s005.pdf]

Supporting Information

Wishes to Die at the End of Life and Subjective Experience of Four Different Typical Dying Trajectories. A Qualitative Interview Study.

Kathrin Ohnsorge, Christoph Rehmann-Sutter, Nina Streeck, Heike Gudat

In the following, we present the original German quotes in the order as they appear in the article. They can be compared with the English translation. Relevant conversational context is included.

Quotes from „4.1. Wish to die and the experience of dying in patients with neurological diseases"

P5-II: "Whether I'd have the courage [pause] *to kill myself, I don't know. But I would genuinely like to fall straight down dead."*

P5-II: Es ist mir verleidet. Es ist mir ganz einfach VERLEIDET! Ich hocke nur noch da. Im Moment darf ich nur für drei Stunden aufstehen *(einatmen).* Auf eine Art und habe ich Dekubitus, oder. Und auf den muss ich auch achten. Und auf das auch. Und dieses muss ich auch noch beachten. Und auf jenes muss jemand anders achten. Und das ist mir einfach verleidet. **Ob ich den MUT hätte, mich umzubringen, das weiss ich nicht. Aber ich würde von Herzen gern sofort tot umfallen.**

P8-II: "Then [during the acute respiratory crisis] I said to my husband, now I want a tablet of some sort and pff! You can't get them [tablets/barbiturates]."

I: Gibt es denn Momente, in denen man sich (.) danach SEHNT? (.) Haben Sie schon Momente gehabt in denen Sie ...

P8-II: Ja (.) also (.) das erste Mal da bei diesem Anfall (*atmet ein*), das war wirklich SEHR erschreckend. (.) Ich habe Panik gehabt. (.) Es sind fünf Ärzte um mich herumgestanden, alle haben diskutiert, was man noch machen kann (..) (*atmet zittrig ein*), haben mir Spritzen gegeben, DAS gegeben, jenes gegeben, gemacht (.) In dem MOMENT (.) habe ich gedacht: (..) jetzt eine Tablette, und pfff, DOCH. (.)

I: Mhm

P8-II: SONST nicht (.), aber in dem Moment

I: Einfach in diesem akuten Moment.

P8-II: Ja

I: Und nachher, wenn (.) die Phase wieder stabiler ist, ist DAS (.) wieder weg, der Gedanke?

P8-II: Ja, dann NICHT, ja (..), ja.

I: Und das war zum: (.) ersten Mal, dass Sie das gedacht haben?

P8-II: Ja (.)

I: Mhm

P8-II: Ja, wenn ich lebe, ist es immer noch SCHÖN, eigentlich (*atmet ein),* aber NEIN, **dort (.), ich habe zu meinem Mann gesagt, jetzt (.) will ich irgend eine TABLETTE und (.) pfff: (.) KRIEGT man ja nicht. Ja.**

I: Und wie, da war wirklich der, also länger dann oder nur dieser EINE Moment, in dem es Ihnen SCHLECHT ging, oder?

P8-II: Also es ist mir ja eben (*atmet ein*) eineinhalb Tage schlecht gegangen.

I: Mh, mhm.

P8-II: In dieser Zeit ist es PERMANENT gewesen (..).

I: Und im Nachhinein? Sind Sie froh, dass Sie diese Tablette nicht bekommen haben,

P8-II: Ich habe gar nicht mehr darüber nachgedacht. Ich habe einfach wieder geatmet, und: ja (..) ja (.).

I: In DEM Moment hätten Sie sie geschluckt (.), wenn es eine gegeben hätte?

P8-II: Das, da hab' (..), es ist wirklich Todesangst, Panik (…) DANN! Ja, dann hätte ich (.) einfach nur (*atmet luftleer ein*), nur stoppen, entweder Luft, oder (..)

I: Und das haben Sie Ihrem Mann auch gesagt?

P8-II: mhm

I: Konnten Sie mit ihm (.) reden, oder?

P8-II: (*in einem kurzen Lachstoss)* Ja, ich hab es ihm einfach an den Kopf geworfen.

I: (*beide lachen kurz*) Ja.

P8-II: Einfach gesagt: (.) er ist kreideweiss geworden, (*atmet ein*) und hat gesagt: hör AUF!

I: (*Lachstoss durch die Nase*)

P8-II: Hab ich gesagt, ja:, ich kriege es ja nicht, ist ja kein Problem (*in kurzem Lachstoss*).

P8-II: "Yes, I think, after a certain point there won't be any more alternatives (…) if even every breath is a burden, so when you can't move in bed on your own, I think, there are situations where that becomes simply unbearable. (…) [if you] can't eat any more, can't speak any more (…) well, then you lie there and get sore from lying, they turn you, hardly have you been turned when – ah – everything hurts."

P8-II: (*holt Luft*) **JA: ich denke, ab einem gewissen Zeitpunkt gibt es auch keine Alternativen mehr (...), wenn nur noch jeder Atemzug eine LAST ist (..), eben wenn man sich nicht mehr allein bewegen kann im Bett (…). ICH denke (..): es gibt Situationen, wo das einfach UNERTRÄGLICH wird. (..) Nicht mehr essen kann, nicht mehr SPRECHEN (…), eben, da LIEGT man und wird wund vom Liegen, (.) man wird gedreht, kaum wird man gedreht, ah, da tut alles weh.**

P8-II: "Then I would say the point has come when, I just have to [say that it] isn't life any longer. […] And if you then help it along a little, I think that's legitimate (…)"

I: Was könnte denn passieren, oder wie könnte es sein, dass Sie, dass Sie sagen würden, jetzt kann ich' s nicht mehr ertragen?

P8-II: Wenn ich mich nicht mehr bewegen kann,- nur noch im Bett liege, und (.) nichts mehr machen kann. (.) nicht mehr essen kann (.) nicht mehr sprechen, (*atmet ein*), das Atmen auch alles je schlimmer und nur noch eine Beatmungsmaske, (.) **dann würde ich sagen, ist der Zeitpunkt, da, wenn es einfach kein Leben mehr ist. (…) Und wenn man DANN ein bisschen nachhilft, denke ich, das ist legitim (..).**

Son of P32-II about the wish to hasten death of his father*: the "image of a total loss of sovereignty"*

S: Und als er / genau, dann hat er das halt / als er die Diagnose bekommen hat, hat er sich dann sehr schnell dort [*Sterbehilfeorganisation*] angemeldet. (.) Weil er einfach gesagt hat: Okay, wenn man sich das so durchliest, was die Perspektive ist, und (.) und letztlich auch, was Frau X. [Ärztin] dann gesagt hat, also, was so / wie die Krankheit verläuft, darauf hat er irgendwie keinen Bock.

I: Mhm (.) Auf was genau hat er keinen Bock?

S: Er hatte keinen Bock darauf, abhängig zu sein. Also/ also, **dieses Bild einer / eines totalen Souveränitätsverlusts.** Weil er einfach (..) also mein / ja, eben, viel Sport gemacht, er lebt alleine zuhause, er hat so seine Rituale, ist einfach sehr selbstständig, ist irgendwie (.) auch selbstgenügsam, und diese Vorstellung, plötzlich von jemandem so abhängig zu sein, gepaart mit den Bildern, die man dann hat, ehm, wenn man z.B. liest was / was da sozusagen / wie das Ende da ausschaut, also auch diese Vorstellung, nicht mehr reden zu können, also an irgendeiner Atmungs- / an einem Atmungsgerät angeschlossen zu sein, Magensonde und so weiter, also diese Vorstellung von einer totalen Anhängigkeit entsprach einfach überhaupt nicht der Vorstellung, oder, von einem Selbstbild, dass er von sich hat. (.) Und ich glaube, diese / dieser / dieser Schrecken dieses Bildes, das ist so das, wo er gesagt hat / und das ist so das, wo wahrscheinlich viele von uns sagen würden: Das will ich auf jeden Fall nicht, diesen Souveränitätsverlust möchte ich nicht. Oder auch diese / diese Abhängigkeit auch von / von einerseits von so vielen Leuten, aber auch das Eintreten in diesen ganzen medizinischen Komplex, diesen Ablauf (.,) ehm, zumal er ja auch / ich meine er ist jetzt [*Ende 70*], also irgendwie so / es ist einfach nicht diese Vorstellung, die er von sich hatte.

P32-II: "If I'm no longer free and can't do anything on my own, I'm totally dependent, then I would probably feel that my life didn't have much meaning anymore."

P32-II: Ja, es ist ja (.) eine Freiheit. Eine Freiheit zu entscheiden was ich machen will, solange ich etwas machen kann, ja. Und wenn ich nichts mehr machen kann, dann kann ich wollen, so viel wie möglich, und es geht nicht mehr. Und dann bin ich nicht mehr frei, etwas zu tun, wo ich entscheide, ja, wo ich einen Willen habe, weil ich ein Bedürfnis habe, oder ich etwas machen muss und so. Und dann / und dann bin ich nicht mehr ich selber. Wenn ich nicht mehr frei etwas tun kann, wenn ich nicht mehr unabhängig bin. Ja gut, gewissermassen wenn ich noch abhängig bin / mit dem Rollator gehen kann ich noch sehr gut, ich muss mich einfach ab und zu hinsetzen, weil meine Muskeln einfach müde werden. Aber ich habe gestaunt, gestern war ich

noch mit der Tochter spazieren, ja, es war schön, ja. **Aber, ehm, wenn ich (.) nicht mehr frei bin und nichts mehr selbständig tun kann, total abhängig bin, dann (.) hätte ich wahrscheinlich das Gefühl, dass mein Leben keinen grossen Sinn mehr hat.** Und dann könnte ich wahrscheinlich, ehm, EXIT in Anspruch nehmen.

I: Mhm. Dann würden Sie es beenden. (.) Ja, (.) eventuell.

P32-II: Eventuell

I: Genau. Ja. (.) So dieser Aspekt, dass / dass Sie anderen zur Last fallen, haben Sie den auch / haben Sie den auch gehabt?

P32-II: Ja, natürlich. Ja.

I: Wirklich?

P32-II: Ja. Ich / ich / also am Anfang sehr. Ich habe auch meinen Kindern gegenüber / eben weil, ehm, die sind verheiratet, haben selbst Kinder, sind professionell tätig und so und für mich einspringen und so, das ist oft, ehm, (.) ja, sie tun es, aber es kostet sie auch etwas zeitlich und so. Gut, sie tun es gerne, ich habe nicht das Gefühl, dass sie irgendwie etwas Obligatorisches absolvieren müssen oder so. Aber ich habe schon ehm / ich möchte schon nicht anderen, der Familie oder meinen Kollegen zur Last fallen. Zum Beispiel, wenn sie so / wenn man ein Fest hat oder Einladungen zum Essen oder so, dann tun sie / wenn man etwas feiert, dann / eine Feier ist doch etwas Freudiges, etwas, das man geniesst, nicht. Und dann hockt da so ein / ein Haufen, der sich nicht mehr bewegen kann und nicht mehr mitmachen kann und so, dann ist das innerhalb von einer Feier eventuell ein negativer Punkt der / „Was hast denn du“ / „Was hat denn der“ und so, und dann ist das ein negativer Einfluss auf die Stimmung irgendwie, und das würde mich schmerzen.

P32-II: “And I don’t really want to waste away miserably. But to leave this world in a state where I can remain somewhere in the memories of my children and my friends and so on as someone who, well, you could talk to and who was reasonably normal.”

P32-II: Und als die Diagnose da war, habe ich mich relativ bald bei der EXIT angemeldet, weil ich wusste, es geht mir immer schlechter, und man hat mich auch gewarnt, dass man bei der EXIT fähig sein muss, selber diese Tasse zum Trinken oder diese venöse Ding, ehm, zumachen, und man muss noch jemanden haben, der bereit ist / einen Arzt, der bereit ist zu unterschreiben, dass man noch zurechnungsfähig ist und so weiter, habe ich gesagt: Gut, ich untersuche das Ganze mal, was es bedeutet. (.) Ja, dann habe ich Unterlagen bekommen, ehm, und ich bin damals / habe ich schon, ehm, viele Defizite gehabt, die sich mehr und mehr ausgebreitet haben. Zum Beispiel im Februar habe ich aufgehört, Auto zu fahren, weil ich einfach nicht mehr die Kraft hatte, das Steuerrad und die Gänge und so weiter zu schalten, das war schon mal eine grosse Einschränkung meiner Bewegungsfreiheit und so weiter, nicht? Und dann noch weitere Sachen, und dann hatte ich so eine gewisse Phase, die ich so als eine Art „EXIT-Phase“ gesehen habe, wo ich einfach das Gefühl hatte / wo ich einfach das Gefühl hatte, das würde mich erleichtern von diesen Beschwerden, die ich habe. **Und ich möchte eigentlich nicht elendiglich versiechen. Sondern diese Welt in einem Zustand verlassen, in der ich für meine Kinder und für meine Freunde und so irgendwie in Erinnerung bleiben werde als jemand, ja, der ansprechbar ist und der einigermassen normal ist.**

P5-II: "How it is now, that's just, for all of it, for wiping your bottom, to pee you need a bag… You just always need help for everything. Recently we went to the council, to sort that out, so that my daughter gets the power of attorney, because of the signatures, yes? I can't sign things any more! I can't even do that!"

P5-II: **Wie es jetzt ist. Das einfach nur noch (.) für alles zusammen (.), für's Hintern-Abwischen, fürs Pinkeln brauchst du einen Sack... Für alles brauchst du immer nur Hilfe. Kürzlich waren wir auf der (..) Gemeinde, um das zu regeln, dass die Tochter (..) die, ehm, Dings hat, die Vollmacht bekommt, wegen den Unterschriften, oder. Ich kann nicht mehr UNTERSCHREIBEN! Nicht mal mehr DAS kann ich!**

P34-II: Some of them said that they were members of a right-to-die organization, so as to be sure of "keeping the last door open" to be able to decide for yourself (P34-II).

P34-II: Ich glaube, das habe ich Ihnen schon das letzte Mal gesagt. Ich bin schon SO lange bei *[der Sterbehilfeorganisation*]. Und ich denke, das wäre für mich ... Also mit meiner Diagnose wäre das sicher kein Problem. Aber ich wollte das eigentlich gar nicht jetzt, oder. Da bin ich meilenweit davon entfernt. Weil, ich habe das dazumal gemacht *[Eintritt bei EXIT],* da ging es mir ja / da war ich schon im Rollstuhl, aber es ist mir viel besser gegangen. Da konnte ich ja wirklich noch alles selber machen. Aber das war für mich / also ich denke - oder ich wünschte mir eigentlich – eben, dass ich nie soweit komme, oder, dass ich zu etwas / oder fände, jetzt kann ich nicht mehr.
Dass ich das gerade lebenslänglich gemacht habe [Beitritt bei Exit]. **Und einfach, um mir die Türe offen zu halten,** obwohl ich nie gedacht habe, oder, da mache ich dann Gebrauch davon, oder dass es mir da psychisch schlechter gegangen wäre oder so. Dass ist überhaupt nicht aus dem heraus entstanden.

P34-II: *„No. I mean, if I realised that something isn't good any more like this [i.e. the dementia in his friend with MS], then I wouldn't want to live any longer."*

P34-II: Einfach weil ich gedacht hatte, weil damals, damals hatte ich einfach gedacht: und wie ist das / ich habe dann immer mehr von anderen gehört /und grosse Schmerzen und habe dann auch den Tod erlebt von Leuten, oder, denen es noch gut gegangen war, wo es dann aber rasant bergab ging und als ich gedacht habe/ eben, einer, bei dem ist das innerhalb von fünf Jahren / der hatte Biologie studiert und als der in die Gruppe kam, hatte er ein bisschen Mühe mit sprechen und nach drei Jahren hat der nicht mehr gewusst, wie er heisst. Und war völlig orientierungslos. Und da habe ich immer gedacht: **Nein. Also wenn ich das merkte, dass etwas so nicht mehr gut ist, dann möchte ich nicht mehr leben. Und das waren eigentlich so diese Überlegungen.**

P8-II explained to her husband that she would want not to be resuscitated in order to spare him and herself years of suffering in which he would have to care for her: “because [*breathes*] there remain saved for me a lot of years of suffering, for me and for you, I said, because he has to look after me, he isn’t so free either then. It took me a long time until he understood it, but I think it got through.”

P8-II: Ja! Total! (…) Ich habe auch geschrieben, zum Beispiel, bei...
I: zur Entlastung?
P8-II: Herzstillstand, ja, keine Reanimation, einfach NICHT!
I: Hmhm
P8-II: Mein Mann ist fast ausgeflippt, (..) und dann habe ich gesagt, nein einfach NICHT, musst es einfach respektierenen, **weil (*holt Luft*) es bleiben mir ein Haufen Jahre Leiden (..) MIR und DIR, habe ich gesagt, erspart, (.) weil ER muss mich ja pflegen, er (.) ist ja auch nicht mehr so frei, dann. (..) Ich habe lange gehabt, bis er es begriffen hat, aber ich glaube (..) es ist durchgedrungen** (..) am Schluss hat er nur gesagt: (*holt Luft*) Du hast so ein gutes Herz, du muss dich gar nicht auf einen Herzstillstand freuen.

P15-II*: The idea of being sent to a nursing home and the blunt conversation made him think “that I would be shut away, like putting the rubbish out.”*

P15-II *(schreibt auf das Tablet, weil sprechen ihm nicht mehr möglich ist)* Jetzt ändert sich das, was ich am meisten fürchtete, nämlich dass ich mit der Krankheit alleine gelassen werde und ich in ein Heim abgeschoben werde. Ich habe einmal gesagt, liebe eine Kugel als ins Heim. […] Aber ein Jahr im Heim und ich bin reif fürs Irrenhaus. Als Wahnsinniger ist das dann egal, was mit mir passiert.
I: Darf ich noch mal nachfragen: Was ist belastend? *(P schreibt, ca. 15 Sekunden Pause.)*
P15-II: **Dass ich weggeschlossen werde *(P schreibt, ca. 40 Sekunden Pause).* Wie Abfall, den man nach draussen stellt.**
I: Hm. (..) Ja, das macht es auch schwer, darüber zu sprechen, das ist völlig verständlich. *(P schreibt, ca. anderthalb Minuten Pause)*
P15-II: Ich weiss, wie es zugeht im Heim. Ich bin nicht der, der Missstände lautlos akzeptiert.

Quotes from 4.2. Wish to die and the experience of dying in patients with organ failure

P11-II: *“I said, I can’t stand this: either die or live, but not this in-between thing.”*

P11-II: Ist seit sechs Wochen so. Nur auf dem Rücken. Was anderes war nicht möglich. Und mein Bauch, also, (..) ich konnte aufstehen, aufs WC gehen, aber sonst nichts. Und dann dachte ich: Vielleicht sterbe ich jetzt. Und da hatte ich das Gefühl, das ist wie

Ebbe und Flut. Das eine ist Leben und das andere ist Sterben, und das wusste ich, das kommt und geht. Kommt nicht drauf an. Ob ich jetzt sterbe, ist das eine, ob ich lebe, ist das andere. Ich überlasse es (...) dem Allmächtigen. Und das habe ich eine Woche erlebt *[nicht leben und nicht sterben können, oder leben und sterben müssen],* und dann habe ich gedacht: Also ich lebe immer noch, wie lange soll das noch gehen? Eigentlich möchte ich sterben. Habe ich schon mit EXIT alles in die Wege geleitet durch meinen Sohn, die Familie war instruiert, einverstanden. **Ich habe gesagt, das halte ich nicht aus: Entweder sterben oder leben, aber nicht dieses Zwischending.**

„P11-II: If I have a relapse, a bad one, then I'll do it [assisted suicide]. Then I'll set it in motion. That's my son, he'll set it in motion.

I: So, what happened, that you decided you want to die now, you want to go to Exit [right to die organisation]? What triggered that?

P11-II: Because I thought, I can't bear it, starting life over another time. Going through it all again. (P11-II)"

P11-II: **Wenn ich einen Rückfall habe, einen schlimmen, dann werde ich es machen. Dann werde ich es in Gang setzen [*den Ablauf für einen assistierten Suizid*]. Das ist mein Sohn, der setzt es in Gang.**

I: **Was war denn da passiert, dass Sie sich entschieden haben, Sie wollen jetzt sterben, Sie wollen jetzt zu EXIT gehen? Was hat das ausgelöst?**

P11-II: **Weil ich dachte, ich halte das nicht aus, noch mal ein Leben anzufangen. Noch mal alles durchzumachen.**

P26-II: *"You know you come to a point, as I said, where you run out of puff […] And afterwards I'll get heart problems, later on. And then they told me, I shouldn't drink so much any more. And previously, with my prostate, it was: 'Drink lots, it'll wash the stuff out!' Because, I always had blood in my water, from time to time, didn't I. And that was the clincher. Afterwards I said: 'Then I've got a problem! If I don't drink anything, down below. If I drink too much, up above. How am I supposed to do that?' Then my doctor said: 'Yes, that will be your problem. In future.' Won't it? And then you start to ponder and think: Why? You could also do it like this. […] And then, it's simple: We would like, if possible, there to be a bit of reality. Everyone has to die. Up to now I haven't found anyone who doesn't. (blows his nose) That's why I say – so – you can be for it, you can be against it"*

P26-II: **Wissen Sie (..), Sie kommen an den Punkt, (..) wie ich gesagt habe, wo Sie keine Luft mehr haben,** wo Sie / wir haben da wegen irgendetwas unterbrochen. Eben – Prostata. **Und nachher bekomme ich Herzbeschwerden später. (.) Und dann (.) haben sie mir hier gesagt, ich dürfe nicht mehr so viel trinken. Und vorher bei der Prostata hat's geheissen: ‚Trinken Sie recht viel, dann schwemmt's das Zeug raus!' (.) Weil, ich habe immer Blut im Wasser gehabt, (.) zwischendurch, nicht. Und DAS hat den Ausschlag gegeben. Nachher habe ich gesagt: ‚Dann habe ich ein Problem! Wenn ich nichts trinke, (..) bei mir unten. Wenn ich zu viel trinke, da oben. Wie mache ich das?' Da sagt mein Arzt: ‚Ja, das wird Ihr Problem sein (.) in Zukunft. (.)**

Nicht?' (.) Und dann fangen Sie an zu überlegen und denken: WARUM? (.) Man kann es auch so rum machen. (.) Das wird also das ewige Thema sein. Nachher hat's geheissen: ‚Wir können Ihnen nur helfen, wenn wir Ihnen die Herzklappe ersetzen. (..) Nachher habe ich gesagt: ‚Wissen Sie, Herr Doktor, wie alt ich bin? (.) Das kommt nicht mehr in Frage. (.) Das hat keinen Wert, wenn wir die Herzklappe ersetzen. Sagt er, ja, das machen wir unten rein, wie ein *Stent*.' (..) Dann habe ich mir das überlegt. Da habe ich gesagt: Ja, dann muss ich ja wieder Medikamente nehmen, um das zu verhindern, dass es abgestossen wird. (.) Und dann hat er mich ein bisschen schräg angeguckt (.) und musste sagen, jawohl. (.) Da habe ich gedacht, ja, da lebt ja der X [*Name von P26-II*] nur noch von Tabletten. Ja, nimmt ja jetzt schon einen Haufen. **Und dann (.) ist es einfach / wir möchten, wenn möglich, ein bisschen eine Realität (.) sein lassen. (.) Es muss jeder Mensch sterben. (.) Bis jetzt habe ich noch keinen (.) gefunden, der nicht ist. *(schneuzt sich)* Darum sage ich (..), die Idee – so – man kann dafür sein, man kann dagegen sein.**

P20-II: *One person who came to the emergency department with an infectious exacerbation of advanced COPD said that the physician at that moment 'broke" his will (P20-II), because he refused to follow his patient's request to stop treatment.*

I: Aha. Mm. Jetzt: Darf ich nochmals zurück kommen auf dieses Gespräch, was Sie da im Spital mit dem Arzt [*über die Situation der Notaufnahme im Moment der Atemnot*] hatten. Ehm, können Sie mir noch mal genauer sagen, WAS Sie ihn da genau gefragt haben?
P20-II: (...) Ich habe gesagt: Ja warum habt Ihr mich nicht sein lassen? Und die Antwort war ganz klar: Ich und wir, wir dürfen das nicht. Sie sind / Ihre Organe sind gesund gewesen, und wir mussten Ihr Leben erhalten, so lange wie wir können. Und dann ist das zweite gekommen, was ich nachträglich gesagt habe: Wenn der Patient so weit entfernt ist von seinem Lebenswillen und dann sagt: Nein, keine Medikamente mehr, speziell keine Antibiotika mehr, ich will nicht mehr, dann können wir Ärzte sagen okay. Und das ist nicht ein Arzt, der das entscheidet, das müssen die Ärzte entscheiden: lassen wir ihn mal so und schauen, was passiert.
I: Und, wenn Sie gesagt haben, also Sie haben ihm gesagt: Warum haben Sie mich nicht sein lassen. Was hätten Sie denn da gerne gewollt in dem Moment?
P20-II: In Ruhe gelassen werden. Ich habe überhaupt von nichts gewusst, was passiert.
I: Also, keine Therapie?
P20-II: Ja.
I: Und auch keine Diagnostik?
P20-II: Nein. Da ist / ich weiss nicht, ob Sie das verstehen ...
I: Doch ich verstehe, ich versuche das zu verstehen.
P20-II: Wenn Sie dort eingeliefert werden, dann sind Sie in einem Zustand, dann möchten Sie von allem nichts wissen. Frag' mich nichts, tu' mir nichts, mach' mir nichts. Aber lass mich jetzt in Ruhe, und lass mich dahingehen.
I: Das erzählen Sie.
P20-II: Das ist der Willen des Patienten.
I: So ging es Ihnen damals?
P20-II: Ja. Und der ist **gebrochen worden** im Prinzip.
I: Mhm. Und wie haben Sie das empfunden, dass der gebrochen wurde?

P20-II: (.) Das ist schwierig zu beantworten. Einerseits ist das / das haben Sie schon auf einer Aufzeichnung von mir, dass ich meiner Frau versprochen habe: Ich bleibe bei Dir, so lange ich kann, weil, sie hängt an mir, und ich hänge an ihr.

I: Mhm.

P20-II: Das hat man jetzt versucht durchzuziehen, und das haben wir jetzt wieder durchgezogen, jetzt bin ich wieder hier seit dem [*Datum*]. (.) Und es geht, aber es ist (..) sehr, sehr mühsam und sehr, es ist, wie soll ich sagen, es ist nicht ein schönes Leben.

P20-II: And he described himself as a candle, saying he was waiting "until the great wind blew me out".

I: Gibt es auch eine alternative Vorstellung die Sie haben? Also einen Wunsch vielleicht oder eine Idee wie es anders sein könnte? Das Ende? Der Übergang?
P20-II: Natürlich. Dass ich auslösche wie eine Kerze, einfach weniger werde. Was man ja hier machen kann. Nicht mehr essen. Aber das geht lange. Das könnte ich mir vorstellen. Das gibt Leute die das machen. […] Ich bin ein Kerzenstock und in mir brennt eine Kerze, um die Kerze weht der Wind, aber der Wind bläst mich nicht aus, weil ich nicht will. Ich will noch leben und ich will machen, was ich noch kann, **bis der grosse Wind mich ausbläst.**"

Quotes from 4.3. Wish to die and the experience of dying in frail elderly patients

P31-II: *Organising the daily routine was becoming more and more "complicated". Even small activities were more demanding. As mobility and concentration decreased, patients tended to organise everything well ahead of time: "Well, I always have to find out more about how I can manage all this. […] I used to be able to cope with it all myself. Just slowly."*

I: Ja. Welche Erfahrungen haben Sie denn mit der Erkrankung in der Zwischenzeit gemacht?

P31-II: **Pfff, ja, dass ich einfach immer mehr herausfinden muss wie ich / wie ich das alles, ehm, managen kann.**

I: Was muss ich darunter verstehen: managen?

P31-II: Also ich meine, ich ziehe mich selber an, ich mache / bin eigentlich bis jetzt im Altersheim noch, ehm, vollkommen selbstständig gewesen. Alles etc., ich konnte noch mit dem Rollator / konnte ich noch Schrittchen für Schrittchen ins Dorf und da dieses Altersheim so toll gelegen ist umgeben von Coop, Apotheke, Drogerie, Blumenladen, ehm, Bank, ehm (.) einfach von allen Geschäften die man braucht, kam ich eigentlich sehr gut davon. **Ich habe das alles immer noch selber bewältigen können. Einfach langsam.**

I: Mhm. Also durch die Erkrankung haben Sie einfach das Tempo verlangsamen müssen?

P31-II: Ja.

I: Ganz viele Tätigkeiten
P31-II: Ja, Tätigkeit praktisch null.
I: Aber so
P31-II: Also Tätigkeit null. Wenn ich//
I: Besorgungen beim / Entschuldigung/ Besorgungen im Coop.
P31-II: Das konnte ich machen.
I: Noch selbstständig.
P31-II: Einfach langsam und dann mit dem Wissen, wenn ich / wenn ich zurück komme bin ich kaputt und dann mag ich nicht mehr und dann kann ich gerade nur noch in / in einen bequemen Stuhl platschen.

P31-II : "And I was just mentally overwhelmed. I'm not as strong as I once was."

P31-II: Es war für mich verrückt. Es war natürlich auch sehr emotional, nicht. Meine Tochter hat mir / ist gekommen und hat mir [*alles*] gepackt und es war ein Zeug und so weiter und ehm dann ist meine Putzfrau, die ist sonst nie gekommen aber dann kommt sie gerade. Ich musste ihr sagen: Hör mal *[Name der Putzfrau]* / Hören Sie mal *[Name der Putzfrau]*, also heute geht es jetzt einfach nicht und immer hat sie weiter geschwatzt und ich habe gesagt: *[Name der Putzfrau]*, es geht heute NICHT! Ich kann nicht mehr. Und dann ja und packen und so und dann ist mir das ja auch nicht (.) ich wusste ja nicht: wo komme ich hin? Es wurde immer nur gerühmt und das Hospiz/ und ich habe NUR Positives gehört.
I: Ja
P31-II: Und da hatte mir schon mal Frau Dr. X. gesagt, ob ich nicht mal 14 Tage raus wolle und wo anders hin. Aber ich wusste ja nicht wie das hier ist.
I: Ja
P31-II: **Und ich bin also einfach / und ich / ich bin einfach psychisch überfordert gewesen. Ich bin nicht mehr so stark wie ich früher gewesen bin**. *(lacht)* und dann bin ich hierher gekommen und habe losgeweint. Weil ich das Gefühl hatte, jetzt darf ich mal weinen. Ich muss nicht die Starke spielen.

P30-II "I thought: well, now, now I can't go on any more. I'm so finished. It's all so complicated inside me."

P30-II: Aber zuerst hatte ich Mühe. Ich habe gedacht: Das ist eine ganz einfache Sache. Ich nehme das Taxi, gehe auf den [*Berg*] und springe runter. *(lacht)*
I: Das haben Sie gedacht?
P30-II: Das habe ich gedacht, ja.
I: Und das war im Zusammenhang mit dem Spital?
P30-II: Mit dem ganzen Theater das ich hatte. Das zu erleben, nicht schlafen zu können, das Schwierige, das hat in mir drin richtig geknorzt wie verrückt.
I: Und das war nach dem Spital? Nach dieser//
P30-II: Auch nach dem Spital habe ich//
I: //Oder schon länger?

P30-II: Das war schon eine Zeit her und ich fand: Das ist doch fertiger Blödsinn, wieso bleibe ich eigentlich hier? Das könnte ich mir doch einfacher machen. Dann habe ich noch gelesen: Man kann ja einfach nicht mehr so viel essen. Das hat Herr Dr. ehm, X aus Zürich mal am Fernsehen gesagt und den schätze ich sehr. [...]
P30-II: Ich habe halt die Meinung: Ich überlasse es einfach dem lieben Gott, er soll machen wie er es richtig findet.
I: Ja. Aber trotzdem war / Gab es da so Momente, wo Sie gedacht haben: Ha, lieber da runter springen?
P30-II: Ja also das, ganz ehrlich gesagt, **ich habe gedacht: Also jetzt, jetzt kann ich einfach nicht mehr. Ich bin so fertig. Alles zusammen ist so kompliziert in mir drin.**

P30-II: *"When you have to relinquish things piece by piece in old age, it's difficult up here. In your head. That's what [bothers me] most, it's really a matter of life. […] If that's life... you're born. You have all these years. And when you go back, all that goes backwards again. And you have to experience so much where you think, no, that can't be possible. […] It's really not easy!"*

P30-II: „Ich habe einfach das Gefühl, der Kopf gehört nicht mehr zu mir. Er ist wie leer. […] Das ist mir durch den Kopf gegangen dort und da habe ich immer gedacht: Also hör mal, das geht doch nicht. Wie kann man das und es gibt Menschen, die viel Schwereres haben als ich. Nicht ich habe es alleine schwer. **Aber wenn man** das ganze Leben nur für andere da war und **im Alter dann so Stück für Stück abgeben muss, hier oben ist es schwierig. Der Kopf. Das ist das was mich am meisten / das ist richtig ein Lebensdings.** Muss sagen, ich habe jetzt Lebenserfahrungen gemacht. **Was das Leben ist, man kommt auf die Welt. Man hat all diese Jahre. Und wenn man zurückgeht, geht das alles wieder so zurück. Und man muss so viel erleben wo man denkt, ja nein, das ist doch nicht möglich.** Jetzt habe ich doch dies getan und ich hatte gedacht ich mache es recht und jetzt geht / geht es mir schlecht und ich weiss mir nicht mehr zu helfen und... **es ist wirklich nicht einfach**!

P30-II: *"I just hope that it doesn't last much longer. It no longer really has meaning for me, you know. I'm not clinging to anything. […] I have no more desires. It's here, but do what you want for all I care. It doesn't mean anything to me any more."*

P30-II: **Ich hoffe einfach, (.) Dass es nicht mehr so lange dauert. Es sagt mir eigentlich auch nichts mehr etwas, wissen Sie. Ich hänge an nichts.**
I: Ah ja.
P30-II: Es gibt nichts, wo ich sagen könnte: Ja, das muss ich haben.
I: Mhm. Und früher war das anders?
P30-II: Ja, früher hat man schon / da fand man dieses schön oder jenes Schmuckstück oder dasjenige fand man besonders. Das verschwindet alles. Also jetzt habe ich es nicht mehr. **Ich habe gar kein Bedürfnis mehr. Es ist hier, aber von mir aus macht was ihr wollt. Es sagt mir nichts mehr.**
I: Mhm

P30-II: Ich bin ein komischer Mensch, Sie entschuldigen. Aber ich bin jetzt so.

P31-II: *"Oh, I'd like to die now. I'd like to die now and I hate the nursing home and the doctor said I should change [homes], but I can't change any more. It's too late. And it's like this in every nursing home. Nowhere is it, nowhere is it, it's more or less better, but um… um… it's like this […] it's not at all easy to exist in a nursing home."*

P31-II: Ich darf's gar nicht sagen. Als ich / jetzt ist es besser, jetzt würde ich es nicht mehr tun. Aber als ich hierher gekommen bin, habe ich gesagt: Und ich will nicht mehr, und ich mag nicht mehr, und äääh, etc. und habe hier rumgenölt etc. und Herr X. hat mit mir gesprochen und / und ... Sehen Sie, bei diesem (.) Wesen, wie es hier ist / (.) wird es dann wieder etwas neutralisiert und wieder ein bisschen und ein bisschen / ein bisschen / man schaut es wieder ein wenig anders an.
I: Ja. Aber dass Sie es dort, kurz nach dem Sie hierher gekommen sind, das hat //
P30-II: //Ja, ich habe gedacht: **Ou, ich möchte jetzt sterben. Ich möchte jetzt sterben und ich hasse das Altersheim und eh, (.) und Frau Dr. hat ja gesagt ich solle wechseln, aber ich kann nicht mehr wechseln. Das ist zu spät. Und es ist in jedem Altersheim so. Es ist nirgends / es ist nirgends / es ist mehr oder weniger besser, aber ehm / ehm, es ist so.** (.) Da hat man halt auch seine / seine / da / **das ist gar nicht einfach in einem Altersheim zu existieren.** (.) Es ist nicht einfach. Ich sag's Ihnen. Das geringste Dings gibt / gibt ehm / ehm, wie soll ich dem sagen, (.)/ es gibt keine Konsequenzen und / und nur schon mal einen Sitzplatz ändern, das ist eine puhh, furchtbare Sache. Eine Zeit lang. Danach bessert es dann wieder. Aber ehm (.) Jetzt habe ich mich daran gewöhnt und ich weiss woher diese bösen / Bösartigkeiten kommen und das wissen alle und ehm (.) und das übersieht man jetzt eigentlich. Eigentlich tut man diese beiden Frauen, die die Bösartigkeit in Person sind, / die sind eigentlich isoliert.
I: Ah ja?
P30-II: Sie sind eigentlich, wie soll ich sagen, selber gestraft.
I: Mhm. Ja
P30-II: Wenn sie sich so abschätzig gegen alle / gegen alle Mitinsassen äussern, oder?
I: Mhm. Ja. Mhm
P30-II: Und das geht auch mal vorüber. Es geht auch beim Personal vorüber und überall vorüber und jetzt steh ich drüber. Jetzt steh ich drüber.

P33-II: *"I've constantly thought, ever since I wasn't so well any more: Oh if only I could just die. Even if only because of the costs."*

I: Was gibt Ihnen grundsätzlich Kraft, das alles auch ehm (.) ehm auszuhalten, oder damit umzugehen?
P33-II: Schwierig. (.) Ich weiss selber nicht. Ich habe immer gedacht das wäre schön, wenn ich sterben könnte.
I: Das haben Sie immer gedacht?
P33-II: **Das habe ich immer /jetzt / seit es mir nicht mehr gut ging, habe ich immer gedacht: Oh, wenn ich nur sterben könnte. Aber schon wegen der Kosten.**

I: Ja. Wegen der Kosten?
P33-II: Ja. auch wegen der Kosten und wegen dem ganzen Umtrieb und dem ganzen Zeug hin und her. Nur darum.
I: Nur deswegen eigentlich?
P33-II: Nur darum. Ja.
I. Ja, mhm
P33-II: Und / Pflege geben ist ja schön, man muss das gar nicht vergessen. Die Leute sagen: Ja, alte Leute kosten viel, Pflege und alles, aber wir geben doch auch viel Arbeit. Oder nicht?
I: He ja.
P33-II: Wir geben doch viel Arbeit für die Pflegerinnen.

P30-II: *"But when you've just been there for others your whole life, and then you have to relinquish things piece by piece in old age, it's difficult. […] well no, what am I here for actually, and the children are grown up, they have their own life, you don't have anything to say any more. So I could just go, I think I could just go. […] I'm… I'm not needed any longer."*

P30-II: **Aber wenn man das ganze Leben nur für andere da war und im Alter dann so Stück für Stück abgeben muss,** hier oben **ist es schwierig.** Der Kopf. Das ist das was mich am meisten / das ist richtig ein Lebensdings. […] Es kommen dann solche Gedanken, das kann kein Mensch weg machen. […] Das geht nicht. Dass man dann sagt: **ja nein, für was bin ich denn eigentlich hier und die Kinder sind erwachsen, die haben ein eigenes Leben da hast du gar nichts mehr zu sagen. Also dann könnte ich doch gehen.** Die haben doch bestimmt noch ein Plätzchen für mich dort oben. *(lacht)* (.) ich muss es auf diese Art nehmen, wissen Sie.
I: Mhm.
P30-II: Das hat mir jetzt darüber hinweg geholfen.
I: Und haben Sie da mal mit jemandem darüber gesprochen?
P30-II: Ja ich habe es auch gesagt, ja. Zu jemandem von der Kirche habe ich gesagt: Weißt du, also ich muss dir ehrlich sagen, ich denke ich könnte doch jetzt gehen. **Ich bin ja / mich braucht es nicht mehr**. Da sagte er: Tante, das tust du mir nicht an! Ich brauche dich noch! Und zwar weil ich mich auch um andere Glaubensgeschwister//
I: Gekümmert habe.
P30-II: Gekümmert habe, enorm. Da hat er gesagt: Weißt du ich brauche das auch noch diese Hilfe, dass man für den anderen da ist wenn etwas ist. Und es ist tatsächlich so.
I: Das ist auch schön zu hören.
P30-II: Ich bin jetzt so.

P33-II: *"I called my son at one or two in the morning, whatever, for him to come and help me go to the toilet. That's always very awkward, I did that for about two weeks. For two weeks I had to call on them at night without wanting to. She's got a household and she goes to work* [daughter in law]*, and it's very awkward for me."*

I: So zum Einstieg möchte ich einfach mal fragen Frau X., wie geht es Ihnen heute?
P33-II: Also ich muss sagen, ich bin ja zuhause / von einem Tag auf den anderen konnte ich nicht mehr selber auf die Toilette gehen. Nicht mehr alleine gehen können, nicht

mehr stehen können. Aus dem Bett und musste mich festhalten, dass ich nicht gefallen bin. Ich bin dann wieder ins Bett, habe die Schwiegertochter angerufen, aber wir wohnen ja nicht im gleichen Haus. Sie ist nebenan. Nicht weit. Auch im Dorf, aber nicht weit. Sie musste jedenfalls immer kommen. **Ich habe nachts um eins oder zwei, ist ja gleich, den Sohn angerufen, dass er kommt und mir hilft auf die Toilette zu gehen. Das ist halt immer sehr unangenehm, das habe ich etwa zwei Wochen lang gemacht.**

I: Zwei Wochen.

P33-II: **Zwei Wochen musste ich sie nachts beanspruchen, ohne es zu wollen. A. hat halt auch einen Haushalt und geht arbeiten und dann ist einem das sehr unangenehm.**

I: Ja. A. ist die Schwägerin?

P33-II: Das ist die Schwiegertochter.

I: Die Schwiegertochter, ja.

P33-II: Man merkt auch wie nett sie sind wenn man Hilfe braucht, und die hat sie mir gegeben.

I: Ja.

P33-II: Nachts habe ich die Nummer auf dem Telefon nicht richtig gesehen und halt an einem fremden Ort angerufen und die hat gesagt: „Da sind Sie aber falsch.“ Da habe ich gesagt: „Ja, ich glaube auch.“ Da sagte sie: „Ja, haben Sie Probleme?“ Diese Frau. Nachts um eins oder um drei, ich weiss nicht mehr. „Ja, haben Sie Probleme? Kann ich Ihnen helfen?“ „Helfen könnten Sie mir schon. Sie könnten mir die Nummer von der Schwiegertochter sagen und sie anrufen, sie soll mich zurück rufen.“ Ist das nicht schön? Da hat sie gesagt das mache sie gerne. Dann habe ich ihr meine Nummer gegeben und sie meinte, eben das sei ja das: Sie habe 80 und ich hätte auf 50 drücken sollen. Das ist ja nur eins drüber.

I: Haben Sie sich da vertippt.

P33-II: Jetzt wo es mir ein bisschen besser geht und ich Dings habe schicke ich ihr eine Karte und danke ihr dafür. Das ist es wert.

Quotes from 4. 4. Wish to die and the experience of dying in patients with cancer

P21-I: *“I can believe it, yes [that someone would feel the need to take their own life]. I came out of the hospital after the diagnosis of cancer and thought, this is what someone who commits suicide feels like.”*

I: Können Sie sich vorstellen, dass es Menschen gibt, die einen Sterbewunsch haben, oder ...?

P21-I: **Das glaube ich schon, ja. Ich bin auch schon aus dem Unispital gekommen von der Diagnose und habe gedacht, so fühlt sich einer, der Suizid macht.**

I: So fühlt sich einer, der Suizid macht.

P21-I: Ja.

I: Ja.

P21-I: Weil das wie ein Hammer kam, oder.

I: Ja.

P21-I: Weil es mir vorher so gut gegangen ist, kam dann der Hammer.

I: Ja. Also das haben Sie auch erlebt (Telefon klingelt), immer wieder so einen Hammer. Ja. (P nimmt den Hörer ab, spricht kurz, legt auf.)

P21-I: Das war meine ältere Tochter. Ich muss auch sagen, ich muss wohl mal einen Moment ausruhen.

Sister of P19-I: *"They [persons with cancer] are people of flesh and blood, with fears, with worries, with uncertainties, up to their neck in catastrophe – so not just the big "C" for "cancer" but also "C" for "catastrophe". [...] There are a lot of worse diseases, but just that word [cancer] ... If a person hears they have cancer, just that alone is devastating, absolutely devastating! For the families as well."*

S: Und ich finde, da müsste man ein viel, viel grösseres Augenmerk darauf legen in der Ausbildung [*auf Kommunikation*], oder wenn es in der Ausbildung nicht geht in der Weiterbildung oder wie auch immer. Ich finde einfach, sie können nicht nur einfach der Mechaniker sein, der das Auto reparieren will, weil es ist kein Auto, es ist kein Blechgegenstand. **Das sind Menschen aus Fleisch und Blut, mit Ängsten, mit Sorgen, mit Unsicherheiten, mit einer riesen Katastrophe am Hals – also nicht nur „K" wie „Krebs", sondern auch „K" wie „Katastrophe", oder.** Und ich finde einfach, das Menschliche bleibt so verrückt auf der Strecke. Und ich finde, das müsste *unbedingt* geändert werden, weil das ist ... **Es gibt einen Haufen schlimmer Krankheiten, aber gerade allein das Wort ... Wenn ein Mensch hört, ich habe Krebs, schon allein das ist *vernichtend*, absolut vernichtend! Auch für Angehörige.**

P2-II: *"Yes, I think especially, if I were to get cancer. [...] That would be a catastrophe for me! [...] For some reason, I have a horror of that. […] You know, there are some things that one just has a horror of, and you can't overcome it. [...] Yes, yes, cancer of course has a bad reputation."*

I: Können Sie sich für die Zukunft eine Situation vorstellen, dass irgendetwas passiert, dass Sie sagen, jetzt möchte ich nicht mehr weiterleben?

P2-II: **Ja, ich denke vor allem, wenn ich irgendwie ein Krebsleiden kriegen würde.** Das habe ich bis jetzt nicht.

I: Was verbinden Sie denn mit einem Krebsleiden, dass Sie sagen, dann nicht mehr?

P2-II: Ja, ein gewisser Horror.

I: Was würde passieren, wenn Sie Krebs bekämen? Wie wäre das? Was wäre anders? Was würde es schlimm machen?

P2-II: **Das wäre für mich eine Katastrophe!**

I: Ja?

P2-II: Ja, ja. Aber ich habe keine Anzeichen für ein Krebsleiden, überhaupt nicht. Das haben wir regelmässig untersuchen lassen. Aber Sie wissen, wie das ist mit Krebs-

Krankheiten, die plötzlich anfangen. Man kann eine akute Leukämie kriegen und da kann man nicht viel machen.

I: Warum finden Sie diese Krankheit so schlimm?

P2-II: Ich weiss es nicht. **Irgendwie hab ich einen Horror davor.**

I: Mhm.

P2-II: Ich denke, das ist auch allgemein so. Leute, die überrascht werden von einer Krebs-Krankheit, und man versucht dann sie (...) vorzubereiten, helfen, das ist sehr, sehr schwierig. Sie brauchen Monate, wenn nicht Jahre, um diese Situation zu akzeptieren.

I: Bei Ihrer Krankheit ist das nicht so?

P2-II: Nein, nein, nein.

I: Was ist da anders?

P2-II: Ich weiss nicht, das ist einfach meine Einstellung. **Wissen Sie, es gibt einfach so Sachen, da hat man einfach einen Horror davor und das kann man nicht überwinden.**

I: Und Krebs ist so etwas.

P2-II: **Ja, ja, Krebs hat natürlich einen schlechten Ruf.**

I: Da sind Sie nicht alleine, das geht vielen Leuten so.

P2-II: (hustet) Hat einen sehr schlechten Ruf. Aber ich hoffe, mir bleibt das erspart.

P5-II: *"I signed up for EXIT, purely for safety reasons, in case I wanted to pull the emergency brake. […] I am signed up to EXIT, I sorted that out almost at the same time as the hospice. EXIT would really just be an emergency handle and not because I particularly wanted it. It would be only if I felt I couldn't get off this train. It wouldn't be because I actually wanted that."*

I: Und zwar fragen wir immer, ob, also bei Menschen mit unaushaltbarem Leiden dürfen Menschen ganz legal einen Dauerschlaf einleiten, eine sogenannte Sedation. Wäre das für Sie eine, eine Möglichkeit, wenn Sie jetzt Ihre Situation nicht mehr aushalten?

P5-I: Ja, das hab ich auch mit der [*Ärztin*] besprochen. Sie hat mir von dem erzählt, und dann hab ich gesagt: Hören Sie, wenn's dann mal soweit ist, wäre ich eigentlich sehr dankbar um das. Dauerschlaf, aber keine Verlängerung dann noch dazu, sondern wirklich dauerschlafen können und die Natur schaffen lassen, aber wirklich, dass ich das wählen könnte. Also nicht, das wäre dann nicht suizidal *(lacht auf),* **ich bin zwar angemeldet, ich hab mich aus, rein aus sicherheits-technischen Gründen hab ich mich bei EXIT angemeldet, wenn ich die Notbremse ziehen wollte.** Also wenn man dem Wunsch nicht folgen würde, dann sag ich dann: *(lacht)* vielleicht hab ich noch die Kraft, EXIT zu rufen. Aber ich möchte das verhindern, dass ich's so aktiv machen muss.

I: Aber trotzdem wäre das (...) also die letzte Möglichkeit?

P5-I: Das ist die Notbremse für mich. Also wenn man mich nicht respektiert, dass ich dann sag, dann würd ich sagen, dann schalt ich das ein. Das wäre der letzte, den letzten alleinige Gang, den mach ich selber. [...]

I: Und dieser Wunsch oder diese Vorstellung die Sie eben beschrieben haben, dass es auch mal irgendwann vielleicht aufhören könnte// haben Sie eben gesagt, Sie würden gerne aufhören wollen?

P5-I: Also nicht, dass ich mir etwas antun wollte, nach wie vor nicht. Aber ich bin sehr aufmerksam und habe die Medien ein wenig verfolgt wegen dieser ganzen *(lacht)* Sterbehilfeangelegenheit. Ich habe gedacht: Huch, jetzt wird es noch schwierig, wenn ich da auf lange Frist hin beweisen muss, dass ich das geäussert habe – wo muss ich das genau deponieren? Wobei ich immer gesagt habe – und das vertrete ich heute wieder – ich habe immer gesagt: **Ich bin bei der Exit angemeldet, das habe ich fast gleichzeitig mit dem Hospiz erledigt. Exit wäre wirklich nur ein Notgriff und nicht, weil ich mir das wünschte. Das wäre nur, wenn ich das Gefühl hätte, dass ich von dieser Bahn nicht mehr runter komme. Das wäre also nicht, weil ich das wollte.** Und auch seit wir uns das letzte Mal gesehen haben, hatte ich nie den Gedanken, mir etwas anzutun. Oder etwas zu provozieren. Dafür lebe ich doch noch zu gerne, oder habe das Leben zu gerne. Da hatte ich nie Zweifel. Ich habe immer noch das Gefühl, die Natur würde schaffen, ich hoffe das auch. Aber wenn es so mühsam ist und ich nicht weiss, wie ich Socken anziehen soll oder es mir so speiübel ist oder ich solche Schmerzen habe, dass ich denke: Mensch huuuuh... dann möchte ich schon, dass es aufhört, einfach dass eine Erleichterung eintritt, einfach dass es aufhört. Und dann denkt man halt ans Sterben, weil jeder Mensch denkt, dass es dann aufhört, ob das dann so ist, weiss ich ja nicht. Ja, es ist mehr dies, einfach eine Befreiung von diesem Zustand.

P17-I: *"You know, I have battled with this stupid cancer for a year and a half. I really tried all the operations, tried all the chemo, and as long as there was even a tiny bit of hope, just the tiniest bit, I still travelled to Lausanne to have an operation, I did everything, but the moment they said, we can't do any more for you, I said, okay, now it's finished, now I can't fight any more, now I don't even want to fight any more, I would like to die now, I'd like to go now. That was quite clear for me."*

P17-I: Ja, nein, es geht mir eigentlich gut. Denn ich habe andere Leute sterben sehen und teilweise in einer furchtbaren Umgebung und ich habe immer gesagt: Ich so nicht. So nicht. Ich bin anständig zur Welt gekommen und ich gehe auch wieder anständig aus dieser Welt. Und ich kann das nicht beeinflussen, ich kann die Geburt nicht beeinflussen, aber den Schluss kann ich wählen, wo ich sterben möchte, Gott sei dank! Und das war nur mein Glück, dass ich das noch machen konnte, dass es auch Platz hatte, dass ich hierher [*Hospiz*] kommen durfte, es geht mir jetzt einfach zu langsam. Ich finde, es ist jetzt, jetzt könnten die da oben mal sagen: Komm jetzt. Ich kann es auch nicht beeinflussen, ich muss einfach warten, bis es an der Zeit ist.

I: Aber manchmal merken Sie, es geht Ihnen fast ein bisschen zu langsam?

P17-I: Mir geht es eindeutig zu langsam! Also ich kam hierher und dachte, zwei, drei Tage, dann ist das vorbei. Ist halt nicht vorbei, obwohl ich ja nichts esse und nichts, aber es ist halt noch nicht vorbei. Aber ich merke schon, dass ich schwächer werde. Also ich merke es am Fernsehen, am Anfang habe ich den ganzen Tag ferngesehen und jetzt mag ich kaum mehr die Nachrichten schauen. Auch telefonieren, ja vorher konnte ich eine Stunde sprechen und jetzt sage ich nach zehn Minuten nur noch ja, mhm, ja. Es ist schon// man merkt es schon. Oder auch wenn ich mich anfasse, nur noch überall Knochen, also das ist ganz klar, das spüre ich schon, also geht da schon etwas in die richtige Richtung.

I: Also in die „richtige Richtung“ wie es für Sie//

P17-I: Es muss, **wissen Sie, ich habe jetzt anderthalb Jahre mit diesem blöden Krebs auf alle Seiten gekämpft. Ich habe wirklich alle Operationen ausprobiert, alle Chemo ausprobiert und solange nur ein kleines bisschen Hoffnung war, nur ein kleinstes Bisschen, ich bin noch bis nach Lausanne gereist um mich operieren zu lassen, ich habe alles gemacht, aber in dem Moment als es hiess, wir können jetzt nichts mehr für sie tun, habe ich gesagt okay, jetzt ist fertig, jetzt mag ich nicht mehr kämpfen, jetzt will ich auch gar nicht mehr kämpfen, ich möchte jetzt sterben, ich möchte jetzt gehen. Das war für mich ganz klar.** Dann habe ich mich zuhause hin gesessen und habe alles geregelt, habe das Testament gemacht, alles aufgeschrieben, die Todesanzeige noch selber verfasst, die Liste, an wen sie verschickt werden muss, nicht dass meine Schwester noch an diesem Kuckuckszeug herumstudieren muss.

P17-I: “*This is no quality of life for me. (…) I just find it a shame that I still have to lie around here for so long. You know, someone else could use this bed. Yes, I mean, I don’t know why this has to be so prolonged*.”

P17-I: Ich finde, das ist nicht würdevoll. Und das ist für die Angehörigen grausam. Nein, das will ich also nicht. Nein, ich möchte schon einen natürlichen Tod, aber er soll bitte etwas schneller kommen. Er soll nicht so langsam kommen wie ein Berner (*lacht*). Ja irgendwann kommt es dann. Und je mehr der Körper verfällt und schwächer wird, also ich denke schon, dass es dann irgendwann schon mal schneller gehen wird. Aber ich weiss es auch nicht, ich bin ja keine Medizinerin, ich lasse das jetzt auf mich zu kommen. Ich will mich jetzt auch gar nicht belasten mit sieben Tagen oder sieben Wochen oder drei Tagen.

I: Vielleicht um diesem Gefühl oder diesem Wunsch, dass es schneller gehen soll nochmals etwas nachzuspüren: Was vermuten Sie, was dahinter ist?

P17-I: Weil es einfach für mich sinnlos ist, hier noch länger herum zu liegen.

I: Bei allem Schönen, was Sie jetzt noch geniessen können...

P17-I:**Das ist für mich keine Lebensqualität.** Am Morgen erwachen/ Es ist ja den ganzen Tag über schön, super Pflege und alles, also nicht, dass ich jetzt undankbar sein will, aber **ich finde es einfach schade, dass ich jetzt hier noch so lange herum liegen muss. Wissen Sie, jemand anderes kann dieses Bett auch brauchen. Ja, also ich weiss jetzt nicht weshalb das noch so herausgezögert werden muss.** Vom Medizinischen her ist es ja klar: Es wird immer weniger und immer weniger, da könnte es auch einfach etwas schneller gehen. Aber irgendjemand lenkt das schon. Ich weiss nicht wer, aber irgendjemand lenkt das. Und ich habe keinen Einfluss darauf. Also mich hat mal eine Schwester gefragt: Essen Sie deshalb bewusst nichts mehr, damit es schneller geht? Da sage ich: nein, dann hätte ich ja Hunger. Und ich bin nicht jemand, der früher hätte abnehmen und Hunger haben können. Mich widert das Essen an. Ich kann das Essen nicht anschauen. Und das heisst, der Körper will das Essen nicht mehr.

P32-I: “*I’ve been really unwell in the last couple of months. And yes, sometimes a good day, but then bad, bad, bad. You know, if you feel ill for days, and you can’t eat anything, if you’re almost throwing up when you just smell food, then I thought, there’s no value in this any more. I just want to go to sleep. [...] I just thought, I can’t go on any more. And the feeling sick doesn’t get any better [...] every day is just torture, isn’t it.*”

I: Können Sie sich eine Situation vorstellen, in der Sie nicht mehr weiterleben möchten?

P32-I: Ja. (weinend) Ja, ich habe am Freitag einen Selbstmordversuch gemacht.

I: Mhm, am Freitag haben Sie einen Selbstmordversuch gemacht?

P32-I: (weinend) Mit Tabletten, aber es war zu wenig. Meine Tochter und mein Schwiegersohn haben mich dann gerettet.

I: Ja. – Haben Sie das schon länger überlegt, dass Sie das machen wollen?

P32-I: Ja, eine Zeit einfach. **Weil, es ist mir halt in den letzten paar Monaten sehr schlecht gegangen. Und halt auch mal einen guten Tag, aber dann wieder schlecht, schlecht, schlecht. Wissen Sie, wenn’s einem tagelang immer schlecht ist, und Sie können nichts mehr essen, wenn Sie fast erbrechen, wenn Sie das Essen nur riechen, da habe ich gedacht, das hat jetzt keinen Wert mehr. Ich will jetzt einfach einschlafen.**

I: Mhm. Da wollten Sie einfach, dass das vorbei ist.

P32-I: Ja. [...]

I: Wann ist zum ersten Mal der Sterbewunsch aufgekommen?

P32-I: Ja, sagen wir, eine Woche, bevor ich es gemacht habe. (Pause) **Ich habe einfach gedacht, jetzt kann ich nicht mehr, oder. Und das wird ja nicht mehr besser mit dem Schlechtsein.** Und dann habe ich mir immer überlegt – es ist jetzt blöd, was ich jetzt wieder sage: Mein Enkel ist jetzt gerade in den Abschlussprüfungen. Dann habe ich immer gedacht, nein, jetzt warte ich, bis er die Prüfungen überstanden hat. Und dann hat er noch die Fahrprüfung gemacht. Und dann habe ich gedacht, das warte ich jetzt auch noch ab. Und als er alles hatte, habe ich gedacht: So, jetzt ist es Zeit, jetzt kann ich gehen. Weil, das hat mich noch interessiert, ob das noch alles gut gegangen ist.

I: Das wollten Sie noch wissen, ob das alles gut geht?

P32-I: Ja, das wollte ich wissen. (Pause) Das ist vielleicht jetzt eine dumme ... (lächelt) Aussage, die ich jetzt mache.

I: Nein, nein. Das ist ja nachvollziehbar. Aber es hat sich in dieser Zeit, in dieser Woche vorher, als Sie gedacht haben, jetzt mache ich das, hat sich nichts im Körperlichen verändert? Dass Sie gemerkt haben, jetzt wird es noch mal ärger?

P32-I: Nein, es ist einfach so: Ich habe schon gemerkt, **es ist jeder Tag einfach eine Tortur, oder.** Das habe ich schon gemerkt. Aber ich habe immer gedacht: Du musst jetzt durchhalten, du musst jetzt durchhalten! Das will ich jetzt unbedingt noch erleben. Und das hat mich jetzt schon noch bis zuletzt am Leben erhalten, oder. Und als ich dann gehört habe, dass alles gut gegangen ist, dann war ich wie befreit und dann habe ich gedacht: So, jetzt ist gut, und jetzt mache ich das.

I: Die Tortur ist eben diese Übelkeit, die Sie gequält hat?

P32-I: Ja. Und ich hatte ja Medikamente, aber das hat ja überhaupt nichts genützt, im Gegenteil, ich habe meistens noch wieder alles erbrochen.

I: Haben Sie sich denn mit jemandem unterhalten über den Sterbewunsch? Haben Sie das jemandem geäussert, bevor Sie das gemacht haben?

P32-I: Also nicht an dem Tag. Ich habe einfach meiner Tochter gesagt und meinem Mann – schon vor längerer Zeit: Ihr müsst mal nicht erschrecken. Wisst ihr, wenn ich mal nicht mehr kann, dann nachher werde ich einfach mal schlafen, dann werde ich einfach genug Tabletten nehmen. Das wusste mein Mann, und das wusste meine Tochter.

I: Aber Sie haben das nicht sonst noch anders angekündigt, als Sie das ...?

P32-I: Nein, ich habe einfach gesagt: Eines Tages ist es einfach so weit, dass ich nicht mehr mag. Und dann werde ich das einfach machen. Dass ihr ja nicht erschreckt und nicht enttäuscht seid über mich!

I: Wie haben denn Ihr Mann und Ihre Tochter darauf reagiert?

P32-I: (weinend) Sie haben beide gesagt, sie verständen mich sehr gut. Sie hatten Verständnis, ja.

P4: "Yes, well, I thought umpteen times, I'd like to have someone from EXIT come, so I signed up, with Dignitas as well, um, because I thought: yes, well, if it becomes so unbearable that, that everyone around me has to hold their nose. That was the worst, I think, then I wanted to, well, break off the exercise."

P4: **Ja, also ich habe x-mal gedacht, ich möchte jemanden von EXIT kommen lassen, auch mich angemeldet, auch sogar bei Dignitas, äh, weil ich gedacht hab: Ja, gut, wenn's dann so unerträglich ist, dass, dass alle Leute um mich herum, die Nase zuhalten müssen. Das ist das Schlimmste gewesen, find ich, dann wollte ich also, abbrechen, die Übung.**

P5-I: *"Well, it started with my partner just disappearing after three weeks, and plunging into a marriage to another woman and nine months later becoming a father. And I had wanted children all my life long."*

P5-1: "*Well, my middle brother, for example, didn't speak to me for a year and avoided contact completely, so that he didn't have to come into contact with me. [...] And when that [the cancer diagnosis] came, he hung up the phone and that was it. [laughs briefly] Well, that's, from my point of view that's really him being overwhelmed."*

P5-I: Also eine Freundin, die beispielsweise als ich ihr's offen gesagt hab [*Tumor-Diagnose*], ist die erste Reaktion gewesen: Jessesgott, wo gehe ich jetzt mit meinen Sorgen hin. Das ist die erste Reaktion gewesen!

I: Ja. Hmhm.

P5-I: Eben so als Beispiel, oder. Im Moment hat mich das wahnsinnig schockiert und ‚mögen' *(CH),* aber ich hab's später verstanden. Sie ist so etwas von ehrlich gewesen, oder? Und wir sind, wir sind vorher über zwanzig Jahre befreundet gewesen und

sind's heute noch. Wir haben das klären können. Aber das hat mir im Moment, hat mir das etwas gezeigt, was ich damals noch nicht verstanden habe und mit dem habe ich mich jetzt ein Weilchen auseinandergesetzt. Also wegen, wo irritiert jetzt wer, wen, wie oder aufgrund von was kommt das zu Stande? So hab ich mir versucht, das zu erklären. Und so hat es natürlich nach und nach Situationen gegeben und gibt es immer wieder.

I: Erleben Sie das überwiegend in ihrem Bekanntenkreis? Eher solche Situationen?

P5-I: Ja.

I: Das die Leute überfordert sind dann?

P5-I: Überfordert oder ganz buchstäblich Reissaus genommen haben. **Also mein mittlerer Bruder hat zum Beispiel ein Jahr nicht mehr geredet und den Kontakt komplett gemieden, dass er mit mir nicht muss in Kontakt kommen.** Und er ist eher noch ein ‚pah', pah', ‚hey', ‚auf-den-Tisch' und ist ein sehr rhetorischer Mensch und ja, gegen Aussen so richtig ein Macher-Typ **und wo das gekommen ist: Telefon aufgehängt und das war's dann. (Lacht kurz auf) Also das ist, ja ist wirklich von mir ausgesehen eine Überforderung, ja.**

I: Wie gehen Sie dann damit um, wenn so etwas passiert?

P5-I: Jetzt kann ich dann, im Moment, also jetzt heut nach, ja man lernt ja dann auch, kann ich das eher angehen und ich hab jetzt auch eher die Möglichkeit, dass ich mir irgendwo eine Hilfe hole. In der ersten Zeit, sagen wir in den ersten zwei Jahren ist es relativ schwierig gewesen. **Also es hat angefangen, dass mein Lebenspartner nach drei Wochen einfach verschwunden ist und gerad in eine Ehe sich gestürzt hat mit einer anderen Frau und neun Monate später Vater geworden ist. Und ich habe mir ein Leben lang Kinder gewünscht.** Also es ist relativ viel Stoff, es hat so schon angefangen, oder. Also ich bin zuerst nur mit so Sachen beschäftigt gewesen und weniger mit meiner Krankheit wirklich. Und so habe ich mich reinwachsen müssen. Und habe versucht, Umgang damit zu finden und den Leuten nicht böse zu sein. Sondern versucht, es zu verstehen. Einzig wenn man versucht, die Anderen zu verstehen und in die Empathie geht, verliert man sich selber und vernachlässigt sich selber. Und das hab ich sehr lange betrieben bis ich plötzlich kommunizieren musste: Hey, wo bleibst du.
